# SMAC mimetic drives microglia phenotype and glioblastoma immune microenvironment

**DOI:** 10.1038/s41419-024-07056-z

**Published:** 2024-09-15

**Authors:** Emmanuel Snacel-Fazy, Aurélie Soubéran, Magali Grange, Kevin Joseph, Carole Colin, Philippe Morando, Hervé Luche, Alessandra Pagano, Sophie Brustlein, Franck Debarbieux, Soline Toutain, Carole Siret, Serge A. van de Pavert, Geneviève Rougon, Dominique Figarella-Branger, Vidhya Madapusi Ravi, Emeline Tabouret, Aurélie Tchoghandjian

**Affiliations:** 1 Aix-Marseille Univ, CNRS, INP, Inst Neurophysiopathol, GlioME Team, Marseille, France; 2 Aix-Marseille Univ, Réseau Préclinique et Translationnel de Recherche en Neuro-Oncologie, Plateforme PETRA“TECH”, Marseille, France; 3 APHM, CHU Timone, Service de Neurooncologie, Marseille, France; 4 Centre d’Immunophénomique (CIPHE), Aix Marseille Université, Inserm, CNRS, Marseille, France; 5 Department of Neurosurgery, Medical Center, University of Freiburg, Freiburg, Germany; 6 3D-Brain Models for Neurodegenerative Diseases, Medical Center, University of Freiburg, Freiburg, Germany; 7 Center of Advanced Surgical Tissue Analysis (CAST), University of Freiburg, Freiburg, Germany; 8 Faculty of Medicine, University of Freiburg, Freiburg, Germany; 9 Aix-Marseille Univ, INSERM, INMED, Turing Center for Living System, Marseille, France; 10 Aix-Marseille Univ, CNRS, INT, Institut de Neurosciences de la Timone, Marseille, France; 11 Aix-Marseille Univ, CNRS, INSERM, CIML, Centre d’Immunologie de Marseille-Luminy, Marseille, France; 12 Aix-Marseille Univ, Réseau Préclinique et Translationnel de Recherche en Neuro-Oncologie, Plateforme PE“TRANSLA”, Marseille, France

**Keywords:** Tumour immunology, Cancer in the nervous system

## Abstract

Tumor-associated macrophages/microglia (TAMs) are highly plastic and heterogeneous immune cells that can be immune-supportive or tumor-supportive depending of the microenvironment. TAMs are the most abundant immune cells in glioblastoma (GB), and play a key role in immunosuppression. Therefore, TAMs reprogramming toward immune-supportive cells is a promising strategy to overcome immunosuppression. By leveraging scRNAseq human GB databases, we identified that Inhibitor of Apoptosis Proteins (IAP) were expressed by TAMs. To investigate their role in TAMs-related immunosuppression, we antagonized IAP using the central nervous system permeant SMAC mimetic GDC-0152 (SMg). On explants and cultured immune cells isolated from human GB samples, SMg modified TAMs activity. We showed that SMg treatment promoted microglia pro-apoptotic and anti-tumoral function *via* caspase-3 pro-inflammatory cleavage and the inhibition of tumoroids growth. Then we designed a relevant immunogenic mouse GB model to decipher the spatio-temporal densities, distribution, phenotypes and function of TAMs with or without SMg treatment. We used 3D imaging techniques, a transgenic mouse with fluorescent TAM subsets and mass cytometry. We confirmed that SMg promoted microglia activation, antigen-presenting function and tumor infiltration. In addition, we observed a remodeling of blood vessels, a decrease in anti-inflammatory macrophages and an increased level of monocytes and their mo-DC progeny. This remodeling of the TAM landscape is associated with an increase in CD8 T cell density and activation. Altogether, these results demonstrated that SMg drives the immunosuppressive basal microglia toward an active phenotype with pro-apoptotic and anti-tumoral function and modifies the GB immune landscape. This identifies IAP as targets of choice for a potential mechanism-based therapeutic strategy and SMg as a promising molecule for this application.

## Introduction

Glioblastoma (GB) is the most common and aggressive primitive brain tumor. Its microenvironment is heterogeneous and dynamic, with a complex cell interplay contributing to immunosuppression and promoting tumor growth. Immune cells constitute up to one half of the tumor mass but their infiltration inside tumors is impaired and vessels dysmorphic [1–3]. Tumor-associated macrophages (TAMs) are the main immune cells in GB [4–6]. TAMs are highly plastic and they adapt to their microenvironment by displaying distinct cellular functions [7–9]. They are commonly considered as pro-tumoral, their density being correlated with GB poor prognosis and relapses [10]. They contribute to immune evasion and immunosuppression leading to T cell paucity and dysfunction [11–13]. Reprograming TAM subsets toward immune-supportive cells is a promising therapeutic approach to improve treatment efficiency however challenging because of their heterogeneity.

TAMs include brain-resident macrophages mostly composed of microglia (TAM-MG) [8, 14], monocytes and monocyte-derived cells (TAM-BDM). Over tumor development, TAM-MG display different states of reactivity characterized by morphological changes, the expression of different cell surface receptors, production of mediators, and phagocytosis making them difficult to distinguish from TAM-BDM. Activated TAM-MG display decreased expression of the physiological core protein TMEM119, and upregulated expression of interferon (IFN), phagocytic/lipidic markers and Major Histocompatibility Complex-II (MHC-II) [7, 8]. Their functions are driven by pro-inflammatory (e.g., iNOS, TNFα, IFN) and anti-inflammatory (e.g., IL-4, IL-10, IL-13, CD206, ARG1) cytokines, impairing or favoring tumor growth [15, 16]. At advanced stages of tumor growth, TAM-BDM densities drastically increase [17–19]. Circulating bone marrow-derived monocytes infiltrate brain parenchyma and differentiate into monocyte-derived dendritic cells (mo-DCs), and monocyte-derived macrophages (mo-Mac anti-inflammatory macrophages) [17, 18, 20].

Reprogramming TAM subsets requires the identification of signaling pathways that drive their dynamic phenotypes, spatio-temporal distribution, and function. The E3 ubiquitin ligases, Inhibitor of Apoptosis proteins (IAPs) are crucial regulators of apoptosis and inflammatory pathways. They are inhibited by second mitochondria-derived activator of caspases (SMAC) and are involved in nuclear factor KAPPA light chain enhancer of activated B cells (NF-κB) and Tumor necrosis factor alpha (TNFα) signaling to modulate tumor immunity [21–23]. In cohorts of GB patients, we previously showed that Inhibitor of Apoptosis Proteins (IAP), cIAP1, cIAP2, XIAP and ML-IAP, were overexpressed, ML-IAP having been correlated with a bad prognosis. We demonstrated that these RING-containing IAP can be antagonized by SMAC mimetic GDC-0152 (SMg) which shows the highest affinity toward ML-IAP and able to penetrate inside glioma tumors [24, 25].

In the present study, we showed that in the GB microenvironment, IAP were all expressed by TAMs. Using SMg, we investigated IAP implication in TAM-related immunosuppression. We examined CD45^+^ immune cells in explants from human GB samples or cultured as monolayers. Analyses of their morphologies, secretomes and phenotypes revealed that IAP inhibition impacted TAMs function and composition. Co-cultures of TAM-MG and tumoroids showed that SMg promoted TAM-MG pro-inflammatory phenotype and anti-tumoral function. To complement our studies on human GB, we analyzed in a relevant immunogenic mouse model [17, 18, 26] how IAP regulate the dynamics of TAM density, spatial distribution, phenotype, and function. 3D and intravital imaging at different tumoral stages showed that SMg promoted TAM-MG reactivity, increased immune cell densities, their intra-tumoral infiltration and a remodeling of blood vessels. Mass cytometry further confirmed the decrease in anti-inflammatory macrophages, and an increase in mo-DC and in CD8 T cell density. Functional analyses revealed that SMg drives basal immunosuppressive TAM-MG towards an immune supporting and anti-tumoral phenotype. Altogether our results shed light on IAP cellular function in human GB and point to a potential mechanism-based therapeutic strategy to reprogram TAM landscape by using SMg.

## Results

### IAP expression and inhibition in human glioblastoma immune cells

**Fig. 1:**
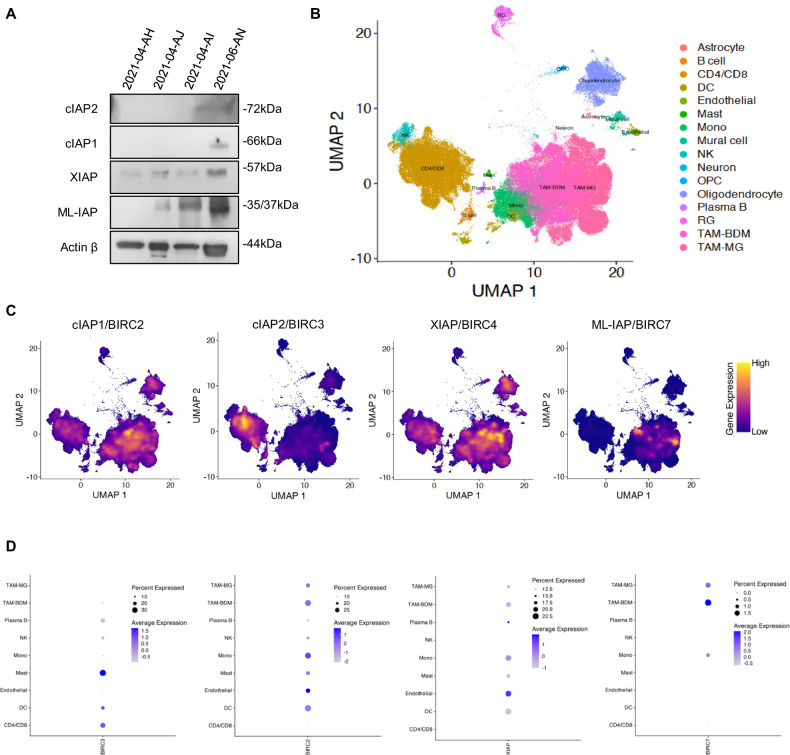
Expression of cIAP1, cIAP2, XIAP and ML-IAP in human GB samples. **A** IAP expression levels were analyzed by Western blotting in hCD45 sorted cells from 4 human GB samples. Expression level of actin β served as loading control. **B** UMAP representing the different clusters of cells composing the GB tumor microenvironment. Each single cell was mapped to previously published states (*n* > 1 million cells). **C** Expression profile of cIAP1/BIRC2, cIAP2/BIRC3, XIAP/BIRC5 and ML-IAP/BIRC7 within the cells of the microenvironment. Note enrichment within TAMs (BDM and MG). **D** Dot plot representation of the expression of cIAP1/BIRC2, cIAP2/BIRC3, XIAP/BIRC5 and ML-IAP/BIRC7 within the cells of the microenvironment. The size of the dots represents the percentage of cells within the population that express the gene of interest. The color of the dots represents the extent of expression within the plots. DC dendritic cells, Mono monocytes, NK natural killer, OPC oligodendrocyte-precursor cells, RG radial glia, TAM-BDM monocyte-derived cells, TAM-MG microglia.

To decipher whether IAP could be involved in GB immunity, we first analyzed their protein and mRNA expression in human GB immune cells. We performed Western blotting of CD45^+^ sorted cells from fresh human GB samples (Fig. 1A). XIAP and ML-IAP were mainly detected. cIAP1 and cIAP2 were faintly expressed in one sample. Then, we leveraged single cell RNA sequencing databases of 110 human GB samples and looked for mRNA expression of ML-IAP/BIRC7, cIAP1/BIRC2, cIAP2/BIRC3 and XIAP/BIRC4 in the immune and endothelial cells clustered in 9 cell subtypes (Fig. 1B) [27]. Concerning the immune cells, ML-IAP/BIRC7 was expressed by TAMs (monocytes, TAM-BDM and TAM-MG), cIAP2/BIRC3 by MAST cells, CD4/8 by T cells and dendritic cells, cIAP1/BIRC2 mainly by dendritic cells and TAMs and XIAP/BIRC4 by TAMs and endothelial cells (Fig. 1C, D). These results demonstrated that human GB immune cells express IAP and that TAM subsets mainly expressed ML-IAP, cIAP1 and XIAP.

**Fig. 2:**
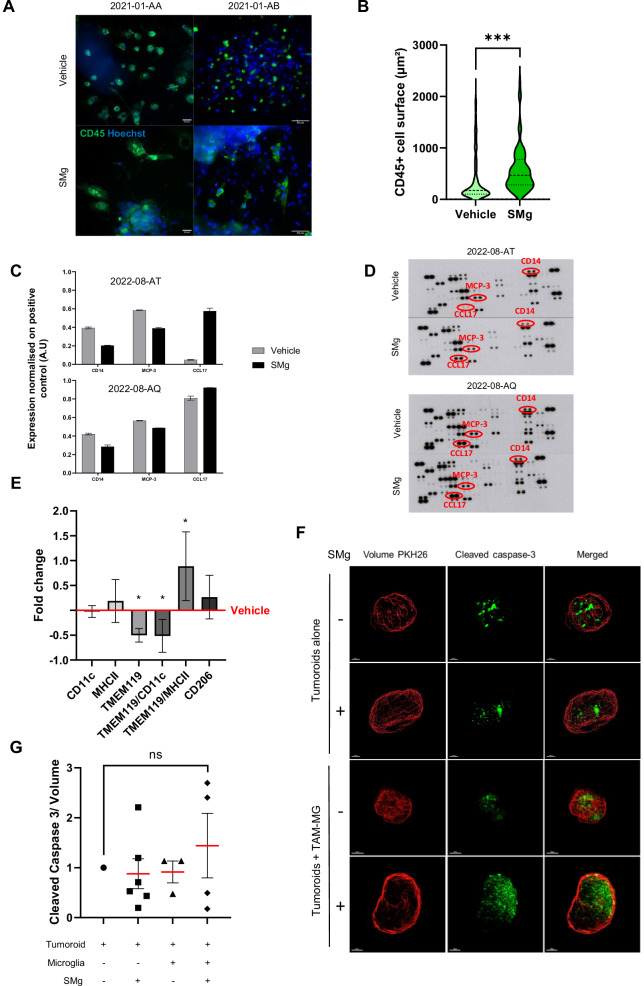
IAP inhibition promotes TAM-MG pro-apoptotic function. **A** Explants derived from human GB were labeled with anti-CD45 antibody (green) and Hoechst (nuclei, blue). Explants were treated for 72 h with vehicle or SMg at 1 µM (scale bar = 50 µm). Representative explants of 1 patient out of 7 are shown. **B** Violin plot of CD45^+^ cells surface (µm²). Quantification was performed with ImageJ, in vehicle (*n* = 311 cells) and SMg-treated (*n* = 123 cells) explants. Statistical analyses were performed by using *t*-test; alpha=0.05, bilateral p-value: ***p < 0.005. **C** Quantification of the main cytokines/chemokines differentially expressed between vehicle and SMg-treated human CD45^+^ cells after 72 h of treatment. The means of duplicates are shown. **D** Membranes of the cytokines and chemokines array. The most significant cytokines/chemokines have been spotted in red. **E** CD45^+^ cells were immunomagnetically sorted from 6 human GB samples. After 72 h of vehicle or SMg treatment at 1 µM, flow cytometry was performed. Fold changes were normalized on vehicle condition. Bar graphs represent mean ± s.e.m. Statistical analyses were performed by using Mann–Whitney test; alpha = 0.05, bilateral *p*-value: *p < 0.05. **F** 3D reconstitution of cleared vehicle or treated tumoroids co-cultured with TAM-MG isolated from the same patient. Tumoroids alone without SMg and without TAM-MG co-culture were considered as controls. Nucview staining (green) identifies caspase-3 cleavage and PKH26 (red) represents the tumoroid volume; Co-Cultures were generated from 3 patient samples. Representative images of a same patient are shown. Scale bar=150 µm. **G** Quantification of caspase-3 cleavage in tumoroids without TAM-MG following vehicle (*n* = 9) or SMg treatment (*n* = 6) and with TAM-MG (vehicle, *n* = 3; SMg, *n* = 4) from 3 human GB samples. Fold changes were normalized on vehicle without TAM-MG condition. Statistical analyses were performed by using Mann–Whitney test; alpha = 0.05. ns non-significant. **C**, **E**, **G** Bar graphs represent mean ± s.e.m.

To determine whether IAP could be involved in immune cell function, we performed explant cultures preserving immune cells in their natural environment and treated them with the IAP antagonist, GDC-0152 (SMg; Fig. S1). SMg modified the immune cells morphology toward a significant increase of their area (Fig. 2A, B) suggesting functional changes. To deeper investigate these changes and better decipher which immune cell subtype could be involved in this effect, we cultured CD45^+^ cells sorted from fresh human GB samples (Fig. S2A, B) and treated them with SMg or with vehicle. After 3 days, a cytokine array was performed from the supernatants. The cytokine profiles were highly similar between patients (Fig. S2C, D). SMg treatment induced variation of three cytokines/chemokines CD14, MCP-3 and TARC1/CCL17 in the same manner between the patients. CD14 and MCP-3 decreased while TARC1/CCL17 increased (Fig. 2C, D). Interestingly, these 3 cytokines/chemokines are known to reflect TAM-MG and TAM-BDM activities [28, 29]. To determine whether their expressions could have a prognosis value, we used TCGA databases to correlate the transcriptomic expression of these 3 cytokines with survival of GB patients. Low level of *CD14* mRNA and high level of *CCL17* mRNA were associated with a better prognosis (Fig. S2D). No correlation was found for *MCP-3* mRNA expression level (data not shown).

Then, to gain some insights in the TAM populations which could be affected by IAP inhibition, we phenotyped CD45^+^ cells sorted from the fresh human GB samples and cultured them in the presence or not of SMg. We used TMEM119 to identify basal TAM-MG, TMEM119/CD11c for TAM-MG in a transitory state of activation and TMEM119/MHCII for activated TAM-MG. TMEM119 is a relevant marker for physiological MG known to decrease when they become reactive [7, 8]. TMEM119^+^ and TMEM119^+^/CD11c^+^ cells were decreased by SMg whereas TMEM119^+^/MHCII^+^ cells increased (Fig. 2E). No tendency was found for CD11c, MHC-II or CD206 alone.

Altogether these results demonstrated that IAP inhibition by SMg modulates TAM phenotypes and TAM-MG activity.

### Function and mechanism of action of IAP inhibition in TAM-MG

We next investigated whether IAP inhibition by SMg could directly modulate TAM-MG function and impact tumor viability and growth. To this end, we set-up a co-culture model composed of a monolayer of TAM-MG TMEM119^+^ cells isolated by FACS from human GB (Fig. S2E) and tumoroids derived from the same patient. The dose of SMg used did not trigger tumoroid apoptosis when they were cultured in the absence of TAM-MG (Fig. 2F, G). In addition, in absence of SMg, TAM-MG did not modify apoptosis level. In comparison, when the co-culture was treated with SMg the level of apoptosis tended to increase in tumoroids (Fig. 2F, G; not significant) showing that TAM-MG is one of the mediator of SMg pro-apoptotic response.

**Fig. 3:**
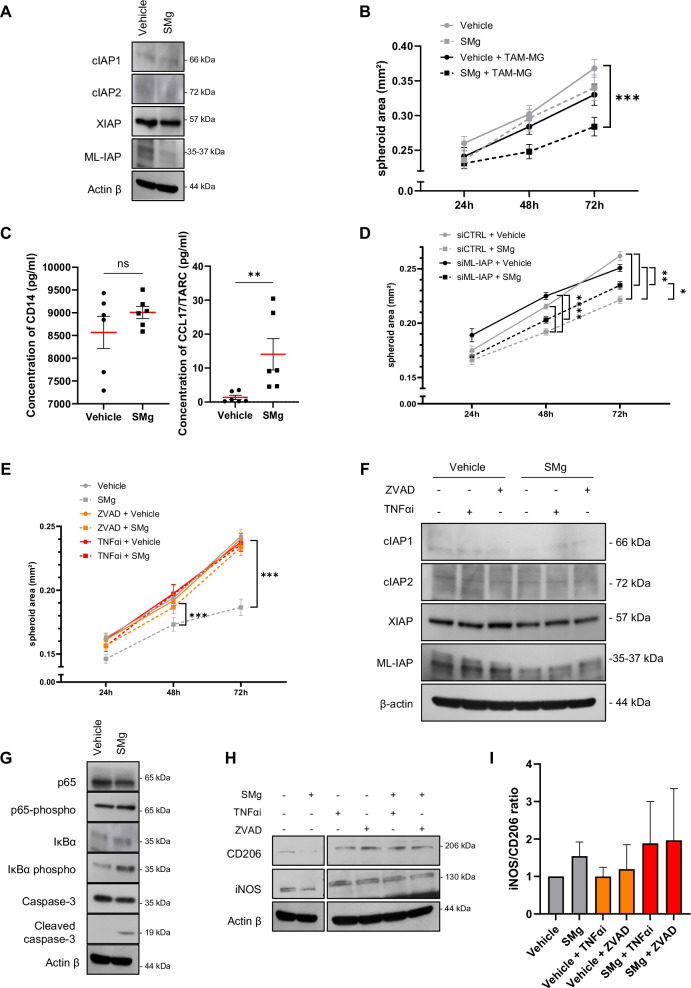
IAP inhibition promotes TAM-MG proinflammatory phenotype. **A** Representative experiment of 3 independent experiments of IAP expression levels analyzed by Western blotting after 72 h of vehicle or SMg treatment in C8B4 microglia cell line. **B** Quantification of GL261-DsRed spheroids area upon vehicle (*n* = 10) and SMg treatment (*n* = 9) and in the presence or not of the C8B4 cells (*n* = 10). Statistical analyses were performed by using ANOVA test and ANOVA post-hoc Tukey test; alpha = 0.05, bilateral *p*-value: ***p < 0.0005. **C** Concentrations of CD14 and CCL17/TARC quantified in the supernatant of C8B4 and GL261-DsRed co-cultures by ELISA assay after 72 h of vehicle or SMg treatments (*n* = 6 independent experiments). Statistical analyses were performed by using Mann–Whitney test; alpha = 0.05, bilateral p-value: **p < 0.005. **D** Quantification of GL261-DsRed spheroids area in the presence of the C8B4 cells expressing ML-IAP (siCTRL) or down-expressing ML-IAP (siML-IAP) (*n* = 10) after 72 h of treatment vehicle (*n* = 10) and SMg treatment (*n* = 9). Data from three independent experiments. Statistical analyses were performed by using ANOVA test and ANOVA post-hoc Tukey test; alpha = 0.05, bilateral *p*-value: *p < 0.05 ; **p < 0.005 ; ****p < 0.0001. **E** Quantification of GL261-DsRed spheroid area in the presence of C8B4 cells treated with vehicle and SMg for 72 h. C8B4 cells were pre-treated for 24 h with ZVAD (vehicle *n* = 23, SMg *n* = 23) and TNFαi (vehicle *n* = 27, SMg *n* = 23) or without pre-treatment (vehicle *n* = 28, SMg *n* = 25). Statistical analyses were performed by using ANOVA test and ANOVA post-hoc Tukey test; alpha = 0.05, bilateral *p*-value: ***p < 0.0005. **F** Representative experiment (*n* = 2) of IAP expression levels analyzed by western blotting after 72 h of vehicle or SMg treatment in C8B4 microglia cell line. C8B4 cells were pre-treated for 24 h with ZVAD and TNFαi. **G** Representative experiment (*n* = 2) of expression levels of p65, phospho-p65, IκBα, phospho IκB, caspase-3, cleaved caspase-3 analyzed by Western blotting in C8B4 microglia cell line after 24 h of vehicle or SMg treatment. **H** Representative experiment out of 3 experiments of expression levels of CD206 and iNOS analyzed by Western blotting after 24 h of vehicle or SMg treatment. C8B4 cells were pre-treated for 24 h with ZVAD and TNFαi. Expression level of actin β served as loading control. **I** iNOS/CD206 ratio in C8B4 microglia cell line after 24 h of vehicle or SMg treatment. C8B4 cells were pre-treated for 24 h with ZVAD and TNFαi. Quantification was performed from 3 independent experiments using ImageJ software and data presented were normalized to actin β expression. iNOS/CD206 ratio fold changes were normalized on vehicle condition. **B**–**E**, **I** Bar graphs represent mean ± s.e.m.

As the TAM-MG isolated from human GB samples could not be expanded in culture and the use of human derived microglia cell lines are not recommended [30], we used the C8B4 mouse microglia cell line to investigate the molecular mechanism involved in this anti-tumoral response. After validating that C8B4 cells expressed IAP (Fig. 3A), we cultured spheroids of GL261-DsRed and CT2A mouse glioma cell lines with or without monolayer of C8B4 cells. The dose of SMg (1 µM) used for the experiments did not impact spheroids size (Fig. S3 A, B) and C8B4 viability (Fig. S3C). At this concentration, SMg decreased mainly ML-IAP expression in the C8B4 cells (Figs. 3A and S3D). The area of GL261-DsRed was not significantly different either when cultured on a C8B4 monolayer or after SMg treatment. By contrast, when the spheroids were co-cultured with both C8B4 and SMg, their area decreased significantly (Fig. 3B). Similar results were obtained with the CT2A cell line (Fig. S3E). This result confirms that microglia is a key mediator of the SMg anti-tumoral effect. We also evaluated a possible paracrine effect of tumor cells on C8B4 cells by submitting them to conditioned medium harvested from GL261-DsRed spheroids and did not observe a significant effect (Fig. S3F). To evaluate whether the cytokine CD14 and the chemokine CCL17/TARC could be a robust readout of this effect as found in human CD45^+^ cells (Fig. 2C, D), we performed ELISA tests from the supernatants of the C8B4 and GL261-DsRed cells co-cultures. Results revealed that CCL17/TARC concentration increased significantly upon SMg treatment while no significant variation in CD14 concentration was quantified (Fig. 3C).

As ML-IAP was strongly expressed by TAMs and specifically by TAM-MG (Figs. 1 and 3A), to decipher whether it could be one of the main actors involved in SMg-response, we designed siRNA targeting ML-IAP. After 24 h of treatment, siRNA against ML-IAP (siML-IAP) decreased ML-IAP expression in the C8B4 cells (Fig. S3G). Then, co-cultures with GL261-DsRed spheroids were performed. Results revealed that in siRNA control conditions (siCTRL), SMg decreased spheroids area (Fig. 3E). In comparison, in siML-IAP conditions, this decrease was partially but significantly impaired (Fig. 3D) revealing the key role of ML-IAP in SMg response in TAM-MG.

As IAP regulate apoptosis and TNFα signaling, C8B4 cells were pre-treated with the pan-caspase inhibitor ZVAD or with a TNFα inhibitor (TNFαi) for 24 h. The medium was removed and the pre-treated C8B4 cells were then co-cultured with glioma spheroids with or without SMg. ZVAD or TNFαi pre-treatments abolished SMg effect on the GL261-DsRed spheroids growth without impairing IAP inhibition (Fig. 3E, F). These pre-treatments decreased also significantly the CT2A spheroids growth (Fig. S3E). The conditioning of C8B4 cells with medium harvested from GL261-DsRed spheroids provided similar results than without conditioning (Fig. S3I). No significant difference was obtained concerning the quantification of CD14 and CCL17/TARC concentration upon ZVAD or TNFαi pre-treatment conditions (data not shown). We then searched whether caspase-3 cleavage could be involved. Immunoblotting performed after 24 h revealed that SMg triggered the cleavage of caspase-3 p19 pro-inflammatory form and its downstream NF-κB activation as shown by increased amounts of phospho-p65 and phospho-IkBα (Fig. 3G). In addition, we analyzed the expression of inflammatory mediators iNOS and CD206. The value of the iNOS/CD206 ratio is indicative of the balance toward a pro-inflammatory or an anti-inflammatory phenotype. Quantification showed that iNOS/CD206 ratio was increased with SMg (Fig. 3H, I). No increase in iNOS/C206 ratio was quantified after ZVAD or TNFαi pre-treatments (Fig. 3H, I).

Taken together these results showed that SMg triggers caspase-3 pro-inflammatory cleavage, NF-κB activation leading to TAM-MG anti-tumoral and pro-apoptotic function.

### CD45^+^ cells density and infiltration in a syngeneic immunocompetent mouse model

**Fig. 4:**
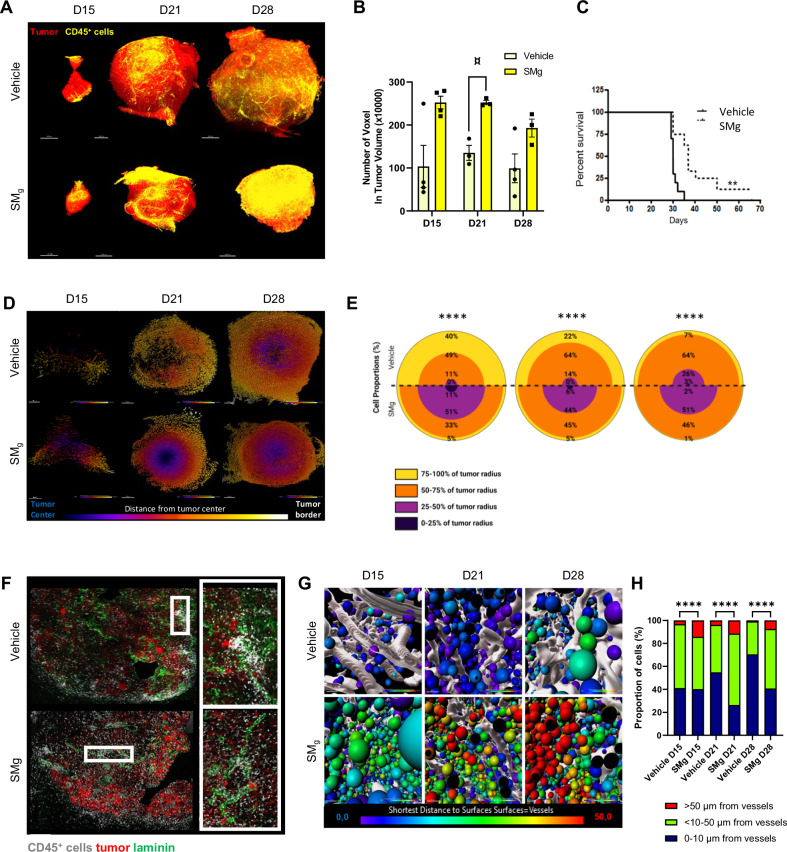
SMg increases CD45^+^ immune cell density and promotes their infiltration. **A** 3D reconstitution of cleared hemi-brains 15, 21 and 28 days-post GL261-DsRed cells graft (D15, D21 and D28) of vehicle (*n* = 11) and SMg-treated (n = 10) mice. Tumor cells were labelled with an anti-RFP antibody (red), immune cells with an anti-CD45 antibody (yellow). Scale bar = 500 µm. Representative images are shown. **B** Quantification of CD45^+^ cell density in tumors (vehicle, D15 *n* = 4, D21 *n* = 3 and D28 *n* = 4; SMg, D15 *n* = 4, D21 *n* = 3 and D28 *n* = 3). Data were normalized to the respective tumor volume. Bar graphs represent mean ± s.e.m. Statistical analyses were performed by using the Kruskal–Wallis test; alpha = 0.05, bilateral *p*-value: D15 vehicle-SMg = 0.083; D21 vehicle-SMg = 0.057; D28 vehicle-SMg = 0.248; ¤ = tendency. **C** Survival curves were estimated by the Kaplan–Meier method (vehicle *n* = 8, SMg *n* = 9); **p-value < 0.005. **D** Heat map representing the repartition of the CD45^+^ cells between tumor border and tumor center. Representative axial images are shown. **E** Quantification of the repartition of the CD45^+^ cells between tumor border and tumor center. For each condition and time points, 2 tumors were pooled for the CD45^+^ cells analysis. D15 (vehicle *n* = 18164, SMg *n* = 14750), D21 (vehicle *n* = 174678, SMg *n* = 243797), D28 (vehicle *n* = 2083336, SMg *n* = 11346604). Data are expressed as % of the total number of CD45^+^ cells. Statistical analyses were performed with Khi² test; alpha = 0.05, bilateral *p*-value: ****p < 0.0001. **F** Confocal images of D28 tumors. and CD45^+^ (white) cells. GL261-DsRed tumor cells appear in red, laminin in green and immune cells in white (scale bar=100 µm). White rectangles represent zoomed area. Representative images are shown. **G** Heat map representing the distance of the CD45^+^ cells from vessels at D15, D21, D28. Reconstituted vessels are represented in white. **H** Quantification of the distance of each CD45^+^ cell from vessels. For each condition and time points, all tumors were pooled for the CD45+ cells analysis (for each time point respectively vehicle and SMg; D15: 5 and 4; D21: 3 and 4; D28: 3 and 3). D15 (vehicle *n* = 271,072, SMg *n* = 169,128), D21 (vehicle *n* = 954,805, SMg *n* = 1,135,285) and D28 (vehicle *n* = 1,003,163, SMg *n* = 3,201,000). Data are expressed as % of the total number of CD45^+^ cells. Statistical analyses were performed with Khi² test; alpha = 0.05, bilateral *p*-value: ****p < 0.0001).

To determine the dynamics of the composition and distribution of immune cells upon IAP inhibition in an integrated living system, we used the GL261 immunogenic mouse model which shows a functional tumoral bloodstream and high similarities with human immunity [7, 31–33]. We grafted GL261-DsRed glioma cells intracranially, and 7 days later we started a weekly SMg treatment (D7; Fig. S4). SMg significantly decreased tumor growth (Figs. 4A and S5A–D) and increased mice survival (Fig. 4C). At D15, D21 and D28 after graft, we performed whole mount immunofluorescences of immune CD45^+^ cells and tumor DsRed^+^ cells of vehicle and SMg-treated hemi-brains. Images were acquired at a resolution allowing to detect the tumor bulk. At each time point, although not reaching significance, a tendency in an increase in CD45^+^ cell density was observed after SMg treatment when compared to vehicle tumors (Fig. 4A, B), correlating with a slower tumor growth and better mouse survival.

We next determined whether this increase in CD45^+^ cell density within tumors could be associated with a modification of cell distribution, as in GB immune cells are often localized at the tumor periphery or in the intra-tumoral perivascular spaces [6]. We performed a cumulative analysis of immune cells based on hierarchical clustering representing the distance of immune cells from the tumor center (Fig. S5E). In vehicle condition, almost 90% of the CD45^+^ cells were quantified at the tumor border (50-100% of tumor radius) and only found within the tumor core at D28. After SMg treatment, 50 to 60% of the CD45^+^ cells were located within the inner part of the tumor (0-50% of tumor radius) with 10% of cells present within the tumor core at D15 (Fig. 4D, E). Spatial repartition analysis of 3D images (Fig. S5F) and CD45 labeling on tissue sections confirmed this promotion of infiltration (Fig. 4F). CD45^+^ cells formed a ring at the tumor border in vehicle-treated tumors and were spread within the tumor with SMg treatment. We also observed as previously described [6] patches of CD45^+^ cells on vessels in vehicle conditions that were missing upon SMg conditions. To better analyze the CD45^+^ cells distribution related to vessels, we imaged the same tumors but at a single cell resolution. We then performed a heatmap representing the distance of the CD45^+^ cells to the vessels. In vehicle-treated tumors, 60 to 70% of immune cells were located close to (<10 µm) or in contact with vessels. In SMg-treated tumors, 50–60% of the CD45^+^ cells were located at least 10 µm away from the vessels (Fig. 4G, H). These results showed that SMg promotes CD45^+^ cells infiltration from perivascular areas into the tumor.

As vessels remodeling can favor immune cells invasion [34], we analyzed the morphology of the tumor vasculature by using in vivo two-photon imaging [34]. In these experiments, vessels were revealed by an intravenous injection of QDot 705. In vehicle condition, vessels appeared numerous, thick and tortuous. In comparison, SMg decreased vascular density during tumor growth (Fig. S6A–C) and vessels appeared straighter and thinner as also shown by CD31 immunohistochemistry (Fig. S6F, G) and ultramicroscopy (Fig. S6H).

Altogether these results showed that SMg increased CD45^+^ immune cell density within the tumor, induced a shift from their peritumoral and perivascular locations to a more diffuse penetration into tumors and contributed to tumor vasculature remodeling.

### Dynamic analysis of TAMs upon SMg

**Fig. 5:**
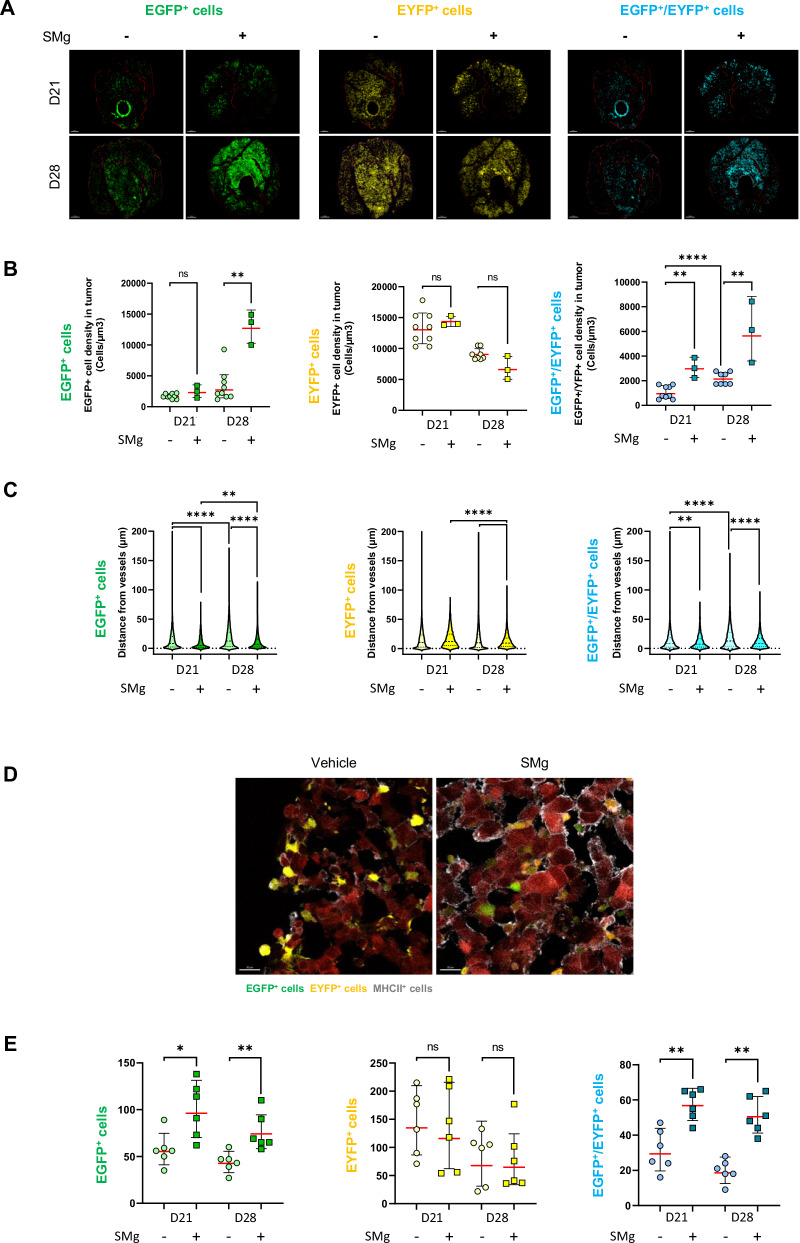
SMg modulates TAM density, spatial distribution and function. **A** Two-photon images at D21 and D28 of EGFP^+^ cells (green), EYFP^+^ cells (yellow) and EGFP^+^/ EYFP^+^ cells (blue). Tumors are outlined in red (scale bar = 300 µm). Representative images are shown. **B** Quantification of EGFP^+^, EYFP^+^ and EGFP^+^/EYFP^+^ cell densities in vehicle (*n* = 9) and SMg (*n* = 3) treated tumors. Statistical analyses were performed by using the Mann-Whitney test; alpha = 0.05, bilateral *p*-value: *p < 0.05; **p < 0.005; ***p < 0.0005; ****p < 0.0001). **C** Violin Plot representing the distance of the EGFP^+^, EYFP^+^ and EGFP^+^/EYFP^+^ cells from vessels at D21 and D28 for each condition. Each dot represents a cell with a distance from tumor vessels expressed in µm. Statistical analyses were performed by using un-paired t-test; alpha = 0.05, bilateral p-value: **p < 0.005; ***p < 0.0005; ****p < 0.0001. **D** Confocal images of EGFP^+^ (green), EYFP^+^ (yellow), MHC-II^+^ (white) cells and GL261-DsRed tumor cells (red) (scale bar = 20 µm). Representative images of D28 tumors are shown. **E** Dot plot representing the number of cells expressing EGFP^+^, EYFP^+^, EGFP^+^/EYFP^+^ and co-expressing MHC-II in vehicle (*n* = 6) and SMg-treated (*n* = 6) tumors at D28. Statistical analyses were performed by using Mann–Whitney test; alpha=0.05, bilateral *p*-value: *=< 0.05; **p < 0.005. **A** 3D reconstitution and image analyses were performed by using Imaris software. **B**, **E** Bar graphs represent mean ± s.e.m.

As we identified a modification of TAM phenotype and activity in human immune cells upon SMg treatment (Fig. 2), we investigated its influence on TAM densities, distribution and function throughout tumor growth. We performed two-photon in vivo microscopy which allows recurrent imaging of a same animal, and we grafted the GL261-Dsred spheroids in triple transgenic fluorescent LysM-EGFP//CD11c-EYFP//Thy1-CFP mouse allowing to identify the different TAM subsets. We previously identified that LysM-EGFP cells are neutrophils and/or monocytes/TAM-BDM (EGFP^+^ cells), CD11c-EYFP are mainly a subset of pre-activated TAM-MG (EYFP^+^ cells), and LysM-EGFP/CD11c-EYFP are mo-DCs (EGFP^+^/EYFP^+^) [17]. In SMg-treated tumors, EGFP^+^ and EGFP^+^/EYFP^+^ cell densities increased significantly whereas EYFP^+^ cells density only tended to decrease (Fig. 5A, B). To decipher if EGFP^+^ cells were monocytes or neutrophils, we performed Ly6G immunostaining on D28 frozen sections to identify neutrophils. The percentage of EGFP^+^/Ly6G^+^ cells was similar for vehicle and SMg-treated tumors suggesting that the increase in EGFP^+^ cells was due to monocytes (Fig. S7A, B).

As we identified a modification of the perivascular distribution of the immune cells with SMg (Fig. 4G, H), we evaluated whether it could alter TAM infiltration in addition to their density by quantifying the distribution of the EGFP^+^, EYFP^+^ and EGFP^+^/EYFP^+^ fluorescent cells relative to the tumor vessels. Without SMg treatment, these cells were always located close to vessels at D21 and D28. By contrast, upon SMg treatment, the EYFP^+^ cells were clustered away from vessels while EGFP^+^ and EGFP^+^/EYFP^+^ cells were mixed in clusters close together (Fig. 5C). We also observed that upon SMg, the morphologies of the EGFP^+^, EYFP^+^ and EGFP^+^/EYFP^+^ cells were modified (Fig. S7C). We quantified these changes and analyzed cell sphericity and cell area to determine if there was a correlation between cell distribution and cell morphology. Independently of the treatment, we noticed that the cells closest to the vessels were the most extended and largest while the most spherical and smallest cells were localized farther away (Fig. S7D). Moreover, upon SMg treatment, EGFP^+^, EYFP^+^ and EGFP^+^/EYFP^+^ cells sphericity decreased and their area increased (Fig. S7E).

We then investigated whether these changes in cell distribution and morphology could reflect changes in cell function. As TAMs can be either immunosuppressive by inducing T cell exhaustion or immunostimulatory by acquiring antigen-presenting cell (APC) function, we performed MHC-II staining to test the TAM antigen presentation ability. We quantified EGFP^+^/MHC-II^+^ cells, EYFP^+^/MHC-II^+^, and EGFP^+^/EYFP^+^/MHC-II^+^ cells on tissue sections at D21 and D28. EGFP^+^/MHC-II^+^ and EGFP^+^/EYFP^+^/MHC-II^+^ cells increased significantly upon SMg treatment supporting a differentiation of the EGFP^+^ cells into mo-DCs with APC function (Fig. 5D, E). No significant difference was recorded for the EYFP^+^/MHC-II^+^ cell subsets. We also quantified EGFP^+^ and EYFP^+^ phagocytic cells which had engulfed red tumor cells and found no significant effect of SMg (data not shown).

Altogether, these results showed that SMg EGFP^+^ monocytes and their derived mo-DCs with APC function, found in mixed clusters, increases the perivascular infiltration of EYFP^+^ cells within tumors while the density of EYFP^+^ cells, which beside pre-activated TAM-MG may also be DCs subsets, is not significantly modified.

### Microglia density, infiltration and function upon SM

**Fig. 6:**
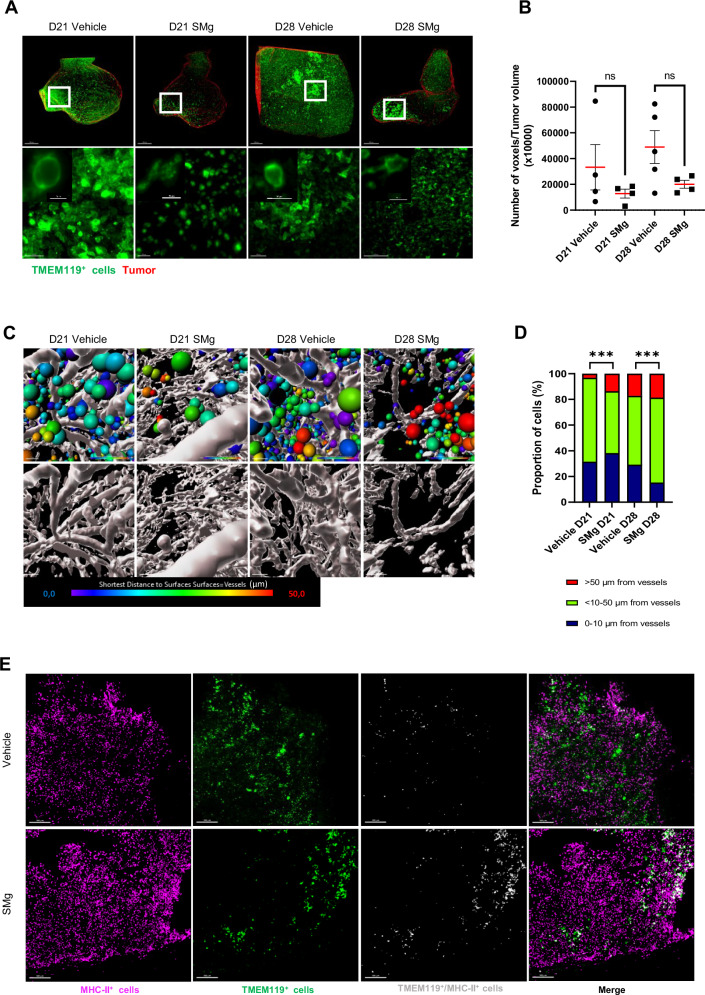
SMg decreases basal TAM-MG density, promote their infiltration and their activation. **A** Representative 3D reconstitution of GL261-DsRed tumors at D21 and D28 in vehicle and SMg-treated mice. Basal TAM-MG were labelled with an anti-TMEM119 antibody (green), and tumors are represented in red (scale bar = 500 µm). White squares identify zoomed areas shown in lower panels. These are Maximum Intensity Projection (MIP) of 1000 µm (scale bar = 100 µm). **B** Quantification of TMEM119^+^ cell densities in vehicle and SMg-treated tumors at D21 (*n* = 4) and D28 (*n* = 4). Bar graphs represent mean ± s.e.m. Statistical analyses were performed by using Mann-Whitney test; alpha = 0.05. **C** Heat map representing the distance of TMEM119^+^ cells from vessels. Each TMEM119^+^ cell (green) is represented by one spot statistically coded by the shortest distance from vessels (heat map: from 0 µm to vessels in blue to >50 µm in red). Representative images are shown. **D** Quantification of distance from vessels for each TMEM119^+^ cell. TMEM119^+^ cells were separated into 3 categories according to their distance from vessels (1 = 0–10 µm, 2 ≥ 10–50 µm, 3 ≥ 50) at D21 (vehicle n = 249602, SMg *n* = 175,272) and D28 (vehicle *n* = 764,564, GDC-0152 *n* = 157,996). Data are expressed as % of the total number of TMEM119^+^ cells. Statistical analyses were performed by using the Khi² test; alpha=0.05, bilateral *p*-value: ***p < 0.0005). **E** Confocal images of TMEM119 and MHC-II co-stainings of D28 tumors (scale bar = 300 µm). Representative images obtained from 2 tumors per condition are shown. **A**, **C** Acquisitions were performed by using 4× optical objective and 2.5× numeric zoom.

To further analyze the basal TAM-MG, as for the phenotyping of the human immune cells, we used the TMEM119 marker. TAM-MG was usually reported to be localized at the tumor border [9, 35, 36]. To gain better information on their amount and organization within the whole tumor, we performed TMEM119 whole-mount immunofluorescences at D21 and D28. In vehicle-treated mice, we observed that the density of TMEM119^+^ cells was highly heterogenous reflecting different states of reactivity. In comparison, upon SMg treatment, all tumors contained a low density of TMEM119^+^ cells (Fig. 6A, B). Moreover, the distance of the TMEM119^+^ cells related to vessels revealed that, compared to vehicle condition, SMg increased the number of TMEM119^+^ cells located further than 50 µm from vessels at D21 and between 10 and 50 µm at D28 (Fig. 6C, D). TMEM119 and MHC-II co-stainings on brain sections identified TMEM119^+^/MHC-II^+^ cells with SMg suggesting that TAM-MG TMEM119^+^ acquire APC function (Fig. 6E).

Taken together these results revealed that SMg decreases basal TAM-MG, promotes TAM-MG tumoral infiltration and modifies their phenotypes towards an APC function.

### TAM-MG and CD8^+^ T cells crosstalk upon SMg

**Fig. 7:**
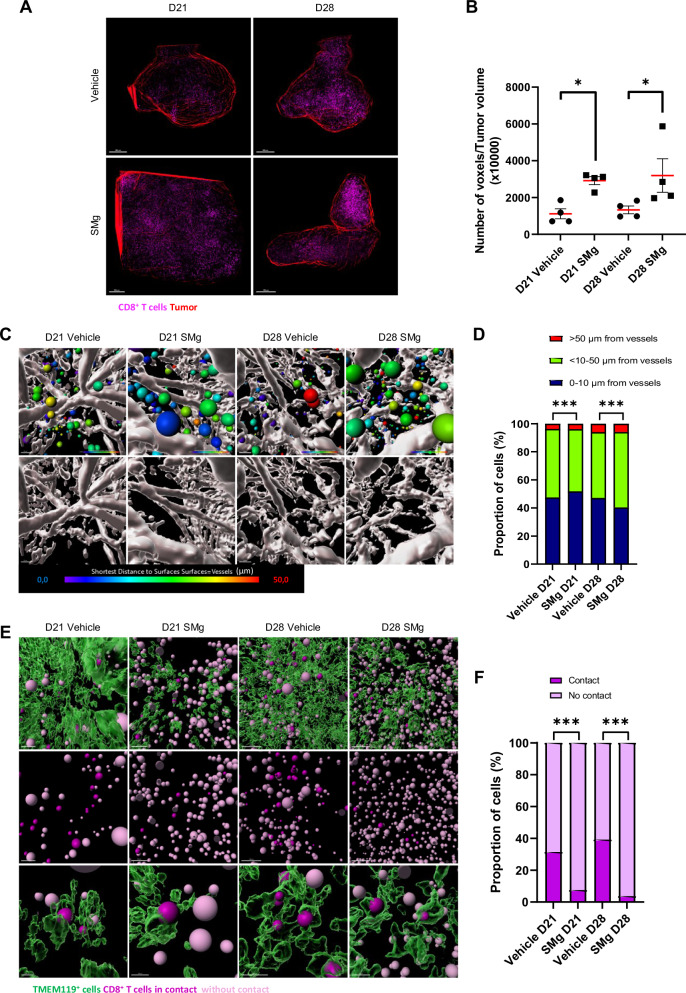
SMg treatment decreases TAM-MG and CD8 T cell crosstalk. **A** Representative 3D reconstitution of CD8^+^ T cells (purple) in GL261-DsRed tumor (red) at D21 and D28 upon vehicle and SMg treatment (scale bar = 500 µm). **B** Quantification of CD8^+^ T cell densities in vehicle and SMg-treated tumors at D21 (*n* = 4) and D28 (*n* = 4). Bar graphs represent mean ± s.e.m. Statistical analyses were performed by using Mann–Whitney test; alpha = 0.05, bilateral *p*-value: *p < 0.05. **C** Heat map representing the distance of CD8^+^ T cells from vessels at D21 and D28. Each CD8^+^ T cell is represented by one spot statistically coded by the shortest distance from vessels (heat map: from 0 µm to vessels in blue to > 50 µm in red). Representative images are shown. **D** Quantification of the distance of each CD8^+^ T cell from vessels. Each CD8^+^ T cells were classified in 3 categories according to their distance from vessels (1 = 0–10 µm, 2 = > 10–50 µm, 3 = > 50) at D21 (vehicle *n* = 21996, SMg *n* = 123,557) and D28 (vehicle *n* = 47952, SMg *n* = 47838). Data were normalized by 100% of total number of CD8^+^ T cells. Statistical analyses were performed by using the Khi² test; alpha=0.05, bilateral *p*-value: ***p < 0.0005). **E** 3D representation of cell contacts between TMEM119^+^ (green) and CD8^+^ T (purple) cells in tumor at D21 and D28 upon vehicle and SMg treatment (scale bar = 100 µm). Representative images of whole mount stainings are shown. **F** Quantification of the TMEM119^+^ cells in contact or not with the CD8^+^ T cells at D21 (vehicle *n* = 33,938, SMg *n* = 152,295) and D28 (vehicle *n* = 90176, SMg *n* = 63,458). Statistical analyses were performed by using the Khi² test; alpha = 0.05, bilateral *p*-value: ***p < 0.0005. **A**, **C**, **E** Acquisitions were performed by using 4× optical objective and 2.5× numeric zoom.

To determine whether this promotion of APC function in TAM-MG could be correlated with their interactions with T cells, we analyzed their crosstalk with CD8 T cells. We performed CD8 whole-mount immunofluorescences at D21 and D28. In vehicle-treated condition, CD8^+^ T cell densities were comparable at D21 and D28. With SMg, their densities increased in tumors both at D21 and D28 (Fig. 7A, B). At D28, the proportion of cells located between 10 and 50 µm and up to 50 µm from vessels increased upon SMg (Fig. 7C, D). Interestingly, in vehicle condition 30% of the CD8^+^ T cells were in contact with the TMEM119^+^ cells and these contacts increased to 40% during tumor growth. Upon SMg, these contacts were significantly lower and dropped over time (7.5% at D21, 3.7% at D28; Fig. 7E, F).

These results showed that SMg promotes CD8^+^ T cell density, infiltration at late stage of tumor growth, and decreases the contacts between TMEM119^+^ and CD8^+^ T cells that could impact CD8 T cell activation.

### Remodeling of the immune microenvironment upon SMg

**Fig. 8:**
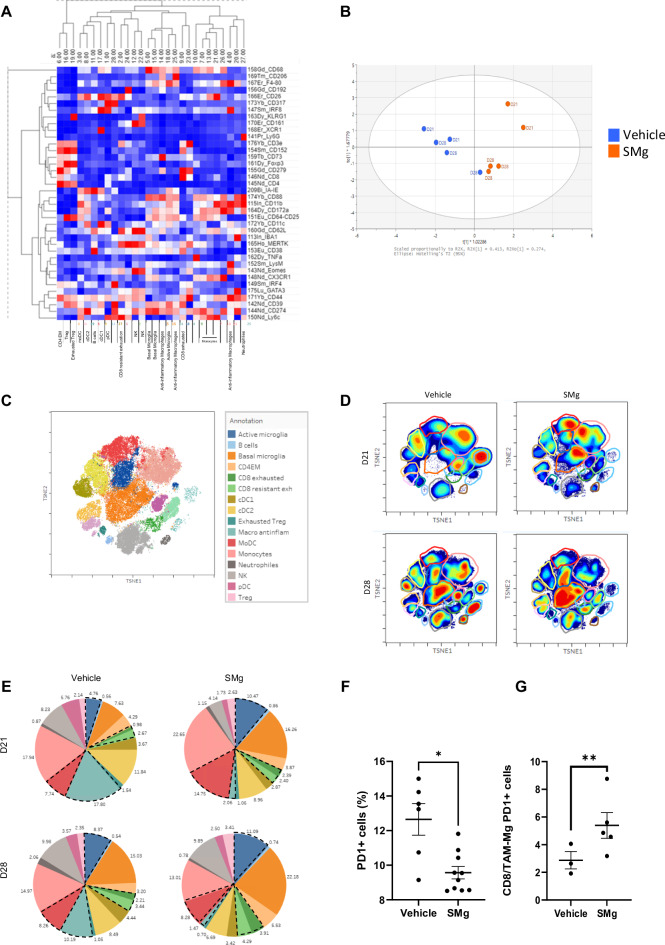
SMg reshapes immune blood-derived cells and brain-resident immune cells. **A** Heatmap representing the level of expression of each antigen in the different immune cell subsets. **B** Multivariate data analysis by SIMCA of vehicle (D21 *n* = 2, D28 *n* = 3) and SMg-treated (D21 *n* = 2, D28 *n* = 3) samples. **C** *t*-SNE unsupervised clustering of each immune population and annotated legend. **D** Heat map of the different immune cell clusters projected onto a *t*-SNE map for vehicle and SMg-treated tumors at D21 and D28. **E** Pie charts demonstrating the distribution of the identified immune cell subsets across conditions. The black dotted lines show the most affected immune cell subsets. **F** Percentage of TAM-MG expressing PD1 among the CD45^+^ cells (basal TAM-MG *n* = 8; active TAM-MG *n* = 8) upon vehicle or SMg treatment. The data from independent CYTOF experiments were pooled as well as those from D21 and D28 conditions. Statistical analyses were performed by using Mann-Whitney test; alpha = 0.05, bilateral p-value: *p < 0.05. **G** Ratio corresponding to CD8 total cells/TAM-MG expressing PD1 cells among the CD45^+^ cells upon vehicle or SMg treatment. The data from independent CYTOF experiments were pooled as well as D21 and D28 conditions (basal TAM-MG *n* = 22; active TAM-MG *n* = 22). Statistical analyses were performed by using Mann-Whitney test; alpha = 0.05, bilateral p-value: **p < 0.005. **F**, **G** Bar graphs represent mean ± s.e.m.

Our data indicate that if TAM-MG seems to be a major population primarily responding to SMg treatment, other subsets of myeloid and lymphoid cells could be impacted, and their function modified. To have a global view of SMg effect on these populations, we performed mass cytometry experiments (CYTOF) at D21 and D28 on sorted CD45^+^ cells. We set up a panel of 38 antibodies used for a total of 5 experiments (Fig. 8A; Table S2). The experiments were analyzed separately as an averaged quantification could not be performed because of the batch effect identified (Fig. S8A). We only considered as major changes the ones that were consistent between the different experiments. Figure 8 presents results of samples that were processed concomitantly. Vehicle and SMg-treated CD45^+^ cells clustered separately (Fig. 8B) and were classified into 16 immune cell subsets which densities were time and treatment-dependent (Fig. 8C, D). For D21 vehicle condition, the main clusters were clusters 13 (monocytes), 4 (anti-inflammatory macrophages), 8 (cDC2), 3 (mo-DCs) and 15 (basal TAM-MG). For D28 vehicle condition, these clusters were conserved in addition to an increase in clusters 15 (basal TAM-MG) and 18 (reactive TAM-MG) and a decrease in cluster 4 (anti-inflammatory macrophages, Fig. S8B). When compared to untreated condition, upon SMg treatment at D21, monocytes, mo-DCs and activated TAM-MG densities increased while anti-inflammatory macrophages and cDC2 were lower in all experiments performed (Figs. 8D, E and S8C, D). At D28, activated TAM-MG, CD4 effector memory (EM), CD8 effector exhausted T cells (CD8 EE) and CD8 resistant to exhaustion T cells (CD8 RE) were increased while anti-inflammatory macrophages were drastically decreased in each experiments performed (Figs 8D, E; and S8E, F).

These results confirmed the increase in mo-DCs observed previously by two-photon imaging and identified a decrease in anti-inflammatory macrophages. We could also clearly establish that SMg increased activated TAM-MG and CD8^+^ T cell subsets.

We finally quantified CD279/PD-1, an inhibitory receptor of immune cells known for its role in tumor immunosuppression, in basal and active TAM-MG with vehicle or SMg treatment. PD-1 levels were significantly lower in activated than in basal TAM-MG (Fig. S8G) and in SMg-treated than in vehicle-treated TAM-MG (Fig. 8F). Moreover, the ratio CD8 total / TAM-MG PD1+ cells increased upon SMg treatment (Fig. 8G). These results suggest that SMg could lead to less immunosuppressive TAM-MG.

## Discussion

Identification of signaling pathways controlling TAM spatio-temporal distribution, plasticity and function remains challenging in GB. In this study, we combined imaging and phenotyping techniques, different integrated levels, to unravel the role of IAP in GB immunity. By using human and mouse-derived models, we showed that SMg remodels TAM subsets toward an anti-tumoral and pro-inflammatory properties, revealing them as key drivers of SMg response. SMg activates TAM-MG, promotes their tumoral infiltration concomitantly with a gain of APC function and a decrease in their immunosuppressive status. This remodeling toward anti-tumoral function is driven by pro-inflammatory caspase-3 activation and TNFα signaling.

TAM-MG are the main brain immune cell type present in GB [7, 17, 33]. In healthy brain, a spectrum of transcriptional states has been identified in addition to a regional heterogeneity [8]. Microglia are known to be the patrolling cells of the brain. When they infiltrate tumors, they can rapidly change their state to become reactive. Here we used TMEM119 and CD11c markers to identify TAM-MG. We observed that upon SMg, TAM-MG increased their reactivity, as indicated by the decrease of the levels of the core microglial marker TMEM119 and density of TMEM119/CD11c cells identified as pre-activated microglia [34]. This increase density of activated TAM-MG was confirmed by the mass cytometry data. Moreover, immunohistochemistry revealed an increased density of TMEM119^+^/MHC-II^+^ cells. Therefore, upon SMg activation, TAM-MG decrease expression of TMEM119, acquire APC function while a subset of TAM-MG is still keeping TMEM119 expression. Our results suggest that IAP inhibition results in a rapid transition of TAM-MG from basal to activated state as indicated by the fact that the densities of the transitory activated TAM-MG CD11c^+^ did not increase upon SMg. The APC function displayed by these TAM-MG make them able to activate other immune cells including T cells [35]. We first examined whether the activation state of TAM-MG influenced interactions with CD8 T cells using proximity/physical contact as a read out. We observed that this activation did not strengthen contacts between TAM-MG and CD8 T cells but in contrary abolished them. These results are in line with the TAM-MG decreased immunosuppressive status upon SMg treatment. Based on these observations, we suggest that activated TAM-MG displaying an APC function are not the immune cells activating the CD8 T cells but more likely participate in the activation of other immune cells.

Two-photon imaging and mass cytometry highlighted a reprogramming of TAM-BDM upon SMg treatment. At the different states of tumor growth examined, anti-inflammatory macrophages drastically decreased upon SMg while mo-DC density increased. This increase could be the consequence of the increase in monocytes recruitment and/or the consequence of the promotion of their maturation towards the mo-DC phenotype. In our fluorescent transgenic mouse mo-DCs co-express CD11c and EGFP a marker of monocytes whose recruitment is increased upon SMg treatment. The role of mo-DCs in GB is not very well documented and appears controversial in tumors in general [36]. Mo-DCs cells are able to acquire APC functions and have been described to be mandatory for a good response to chemotherapy or immunotherapy to activate properly the CD8 T^+^ cells [37, 38]. In contrast, they can differentiate into immunosuppressive cells which fail to cross-present tumor antigen and impair T cell proliferation [39–41]. Our data also revealed that mo-DCs increased their expression of MHC-II with SMg, allowing them to actively participate in CD8 T cell activity. In support, CD11c^+^ cells including mo-DCs have been described to excel in their APC capacity contrary to CD11c^+^ microglia subset unable to induce a proper adaptative immune response [42]. In addition, whether activated TAM-MG MHC-II^+^ could activate monocyte maturation and/or signaling to mo-DCs would be interesting to determine.

We also interrogated whether this remodeling of the TAM-MG phenotype could be causally linked to a change in anti-tumoral function. We showed that the TAM-MG anti-tumoral functions acquired *via* IAP inhibition can directly regulate tumor growth. We identified that SMg triggered TAM-MG to acquire pro-inflammatory functions by increasing iNOS/CD206 ratio. This change was dependent of the caspase-3 p19 activation as caspases inhibition blocked this phenomenon. A previous report showed that the switch between pro-inflammatory and pro-apoptotic caspase-3 cleavage in microglia is controlled by cIAP2 [43]. In our experiments, cIAP2 was hardly detected in the CD45^+^ cells isolated from human GB samples or in the TAM-MG. Single-cell RNAseq database analyses confirmed that cIAP2 was not expressed by TAMs but rather by T cell subsets. Therefore, SMg might have a direct effect on T cells via cIAP2 inhibition. Additionally, scRNASeq data highlighted that XIAP/BIRC4 was expressed by endothelial cells and cIAP1/BIRC2. SMg could have a direct effect on tumor vasculature. In comparison we showed that ML-IAP, only expressed by TAMs and strongly in TAM-MG, is one of the main actor of the effect of SMg on TAM-MG remodeling. Therefore, the effects observed on the adaptative immunity and vascular remodeling could rather depend of the inhibition on cIAP2, cIAP1 and XIAP instead of ML-IAP.

The level of penetration of the immune cells inside solid tumors is another major aspect to overcome. In GB, they have been reported to be located at the tumor periphery or in the intra-tumoral perivascular spaces [6, 9, 31, 32]. Our 3D approach revealed that SMg promotes their spreading and infiltration within tumors. They were located more distantly to the vessels. We also noted a vascular remodeling upon SMg treatment. This could explain, at least in part, the higher density of monocytes and T cells coming from the bloodstream. This is also in line with the reported association between vessel normalization and TAMs reprogramming that could be indirectly triggered by IAP inhibition in TAMs [3, 44]. Nevertheless, whether these vessels after SMg pharmacological remodeling could be fully functional, e.g., expressing adhesion molecules as ICAM-1 and ICAM-2, and whether they have been normalized was not further investigated.

The remodeling of the GB microenvironment landscape including immune cells and vasculature could be highly advantageous for clinical applications. Here, we showed that SMg treatment affect both tumor component. SMg drives microglia toward an active phenotype with pro-apoptotic and anti-tumoral function and modifies the GB immune landscape. This identifies SMg as a molecule of choice for TAMs reprogramming that might impact clinical outcome.

## Material and methods

### Human glioblastoma samples

GB tumor specimens were collected at Assistance Publique-Hôpitaux de Marseille (AP-HM) after surgery and placed in Hank’s Balanced Salt Solution (HBSS, Table S1). Samples were obtained from the center of biological resources of AP-HM (CRB BB-0033-00097) according to a protocol approved by the local institutional review board and ethics committee (2014-A00585–42) and conducted according to national regulations. The study was performed in accordance with the declaration of Helsinki. All the patients provided written informed consent. All samples were processed within the 4–24 h following surgery.

### CD45 and TMEM119 cell sortings

Tumors were dissected, automatically sectioned using a McIlwain tissue chopper and enzymatically dissociated with 5 mg/mL of Trypsin (Sigma Aldrich, Paris, France) and 200 U/mL of DNAse (Sigma-Aldrich) for 10 min at 37 °C. Trypan blue staining confirmed more than 80% cell viability. Cells were processed and stained as previously described ([45]; see Table S1). CD45^+^ cells were sorted by MACS as previously described [45], and TMEM119^+^ cells were sorted by FACS (cytoflex, Beckman coulter). Cells were collected and counted for further experiments.

### Cell culture

C8B4 cells, a C57BL/6 murine microglia cell line (ATCC® CRL-2540) were cultured as monolayers in DMEM (ATCC, 30–2002) supplemented with 10% Fetal Calf Serum and 100 IU/ml sodium penicillin G and 100 µg/ml streptomycin sulfate. Cells were kept at 37 °C in a 5% CO_2_ atmosphere.

Human GB CD45^+^ cells were plated on coverslips coated with poly-L-lysine. Two hundred thousand cells were seeded per well in serum-free medium and explants of the same tumor were cultivated in a transwell inserted on the top of the CD45 culture (10 explants/well). After 4 days, immune cells were treated with DMSO or with 1 µM of SMg for 72 h and then washed with PBS before further processing.

#### Mouse glioma spheroids and microglia C8B4 cells co-culture

C8B4 cells were cultured as monolayer in 24-well plate at a concentration of 100,000 cells/ well. The C8B4 cells were cultivated with or without conditioned medium of 4 days old GL261-DsRed or CT2A cells with or without pan-caspases inhibitor ZVAD (1 µM) and/or inhibitor of TNFα (Etanercept (Erelzi®, Sandoz + 100 µg/ml) for 24 h. After 24 h, the medium was removed and one spheroid of GL261-DsRed or CT2A was implanted. Then fresh medium with DMSO or sublethal dose of SMg (1 µM) was carefully added in the wells. Co-cultures were imaged each 24 h for 72 h with a Zeiss Observer Z1. Mosaic acquisitions were performed.

Spheroids were segmented on raw images with Fiji (ImageJ), then area was measured. Area was calculated by multiplying measured area in pixels by 0.73139.10^−6^ (area of a pixel = ${(\frac{{\rm{scale}}{\rm{value}}}{{\rm{scale}}{\rm{value}}{\rm{in}}{\rm{pixels}}})}^{2}$ to obtain a value in mm^2^.

#### Tumoroids and TMEM119^+^ cells co-cultures

Two GB tissue samples were collected in HBSS and processed within 24 h after surgery. Samples were cut twice perpendicularly with tissue chopper to obtain pieces around 0.5mm^3^. Then, the tissue pieces were grown in 6-well plates in 2 ml of serum-free medium supplemented with B27, EGF and bFGF under shaking at 1200 rpm. Tumoroids were then manually selected when their edges were smooth and their cell density was estimated by Hoechst nuclei labeling (1/1000). The selected tumoroids were labeled using a lipophilic dye PKH26Red Fluorescent Cell Linker Kit (Sigma-Aldrich) and cultivated with NucView™ 488 caspase-3 substrate to monitor in live capase-3 activity (Apoptosis Assay Kit NucView™ 488, Biotium, Inc., Calif., USA).

### Cytokine array and ELISA tests

Supernatants were collected after 72 h of treatment and processed using the proteome profiler human XL cytokine array kit (R&D systems, #ARY022B) or the ELISA kits Quantikine ELISA Mouse CCL17/TARC Immunoassay (R&D systems, MCC170) and Mouse CD14 Immunoassay (R&D systems, MC140) accordingly to the manufacturer protocol (Data represented were normalized to reference spot expression in array membrane).

### Flow cytometry

Cells were dissociated with accumax (Sigma-Aldrich) for 10 min at 37 °C. Cells were incubated with Viobility dye (405/452, Miltenyi Biotec) for 20 min in dark. Cells were washed with PBS/BSA 0.5%/EDTA 2 mM and incubated with TMEM119, MHC-II, CD11c, CD11b and CD206 (see Table S1). Flow cytometry was performed using MACSQuant10 (Miltenyi Biotec). Data were analyzed using Kaluza (Beckman Coulter).

### Glioma mouse models

All experimental procedures and animal care were carried out in accordance with the guidelines of the French Government, reviewed and approved by the Regional Institutional Committee for Ethics on Animal Experiments under the authorization number 22184-2019092410097285 and 21353-2019070411491375. C57BL/6 mice (*n* = 97) were housed in cages with food and water ad libitum in a 12 h light/dark cycle at 22 ± 1 °C. A minimum of 3 animals per group were used depending on the experiment. Each group of animals injected with glioma cells simultaneously was divided into two subgroups: one receiving the vehicle treatment and the other receiving SMg treatment.

### Light sheet imaging

Samples were imaged in MACS Imaging solution (Miltenyi Biotec) in sagittal orientation with the Ultramicroscope Blaze^TM^ (Miltenyi Biotec) equipped with a 4.2 Megapixel sCMOS camera and 1.1×/4×/12× objectives. A numerical aperture of 0.1/0.35/0.53 were used with a fixed 4-sources illumination. The microscope is equipped with LED lasers (488 , 561 and 639 nm). Emission filters used were 525/50, 595/40, 680/30. The samples were scanned with sheet of 4 µm of thickness (for 1× and 4× objectives). A horizontal dynamic correction was applied when mosaics were performed with the 4× objective.

### Mass cytometry

#### Cell extraction for Mass cytometry staining

Tumors were extracted (D21 vehicle *n* = 22, SMg *n* = 23; D28 Vehicle *n* = 4, SMg *n* = 6) and minced with curved scissors in 1,5 mL tube containing 300 µL of HBSS/2% (v/v) fetal calf serum (FCS) and Protein inhibitor cocktail (ThermoScientific, #004-980-93), then dissociated using Tumor dissociation kit (Miltenyi Biotec, #130-096-730) at 300 rpm for 10 min at room temperature. Reactions were then stopped using PBS/EDTA (0.1 M). Cells were washed in HBSS/2% (v/v) FCS and Fc receptors blocked using 24G2 hybridoma for 10 min at 4 °C prior to CD45 barcoding.

#### CD45 barcoding and MACS enrichment

Cells were barcoded with a combination of 3 different CD45.2 Cadmium (see Table S2) in Cell Staining Buffer (Standard Biotools #201068) for 30 min at 4 °C. Samples were pooled according to the treatment. Before CD45^+^ enrichment, 10 µL of pooled cells were stained with anti-mouse CD45.2-APC and Ter119-PE for 10 min and counted on Attune NxT Cytometer (ThermoScientific). Barcoded cells were enriched using CD45 microbeads (Miltenyi Biotech, #130-052-301) following manufacturer on a MultiMACS Cell24 separator (Miltenyi Biotech). CD45^+^ Cell enrichment was determined on Attune NxT Cytometer with anti-mouse CD45.2-APC and Ter119-PE

#### Mass cytometry staining

Enriched cells were stained with 100 µL CisPt_198_ (Standard Biotools, #201198) for dead cell exclusion. Cells were stained with 35 µL of anti-chemokine mix (MERTK, CD192, CX3CR1) for 30 mi. Then, 65 µL of mix containing remaining surface antibodies was added and incubated for further 30 min at 4 °C. Intracellular antibody staining was then performed using Foxp3/Transcription Factor Staining Buffer Set following manufacturer recommendations (ThermoScientific, #00-5523-00). DNA was stained overnight at 4 °C with 100 µL Cell-ID Intercalator Ir (201192B Standard Biotools) diluted at 1/1000 in Cytofix/cytoperm (BD 51-2090KZ). On following day, cells were washed twice with 1 mL CSB and pellet was resuspended in 100 µL FCS + 10% (v/v) DMSO and stored at 80 °C until acquisition. Prior to acquisition, cells were washed in water and diluted to 5 × 10^6^ cells/ml in H_2_O containing 10%(v/v) EQ Four Element Calibration Beads (Standard Biotools, #201078). Flow cytometry files (FCS) were normalized to EQ beads signal using standard biotools algorithm and beads were excluded using ^165^Ho versus ^153^Eu. Singlets Cell events were defined using Gaussian parameters, Event length, DNA (^191^Ir and ^193^Ir) channels and Cis^198^Pt negative staining on Cytobank software (Beckman Coulter, www.cytobank.org).

#### Debarcoding and multi-dimensional data analysis

Data were debarcoded using in house developed R-shiny interface ‘Vaevictis’ (https://github.com/stuchly/vaevictis) to perform dimensionality reduction. Debarcoding was done manually on Cytobank using Vaevictis dimensions. Debarcoded files were analyzed using either OptSNE (perplexity = 30 and iterations = 1000) and Rphenograph [46] on ‘CIPHEBank’, a in house developed R-shiny interface to classify and visualize the subpopulations of tumor-infiltrating lymphocytes (TILs) based on the cell surface and intracellular markers (see Table S2). RphenoGraph first identified the k-nearest neighbors (*k* = 30) using Euclidean distance, and calculated the similarities using the Jaccard coefficient. Subsequently, the Louvain algorithm was used to partition the network for detecting communities with optimal modularity, generating 28 metaclusters (Fig. 8). Median expression of marker intensities for each cluster were used for expert-guided manual annotation. Heatmaps and hierarchical clustering were generated using Morpheus (https://software.broadinstitute.org/morpheus). Cluster data were visualized in Cytobank to generate density plot and cluster overlays.

### Image analyses

#### Light sheet imaging

3D projections were performed using Imaris software (Bitplane, http://www.bitplane.com/imaris/imaris). The volume of each tumor or tumoroids was defined by creating a 3D mask using the surface tool from Imaris software v10.0 (Bitplane). For CD45/Vessels/GL261DsRed panel, DsRed anti-RFP labeling was used to determine tumor mass. For Vessels/TMEM119/CD8 panel, autofluorescence obtained with the 488 wavelength was used to determine tumor mass. Both segmentation were compared to validate tumor segmentation (Fig. S10). Only cells present in the tumor mask were segmented according to the local intensity contrast. For brains analysis, to access cell density, the numbers of CD45^+^, CD8^+^ and TMEM119^+^ cells was reported to the tumor volume. To study the repartition of CD45^+^ cells within tumor, each CD45^+^ cell was represented by one spot statistically coded by the shortest distance from tumor center normalized by the tumor size (heat map: diameter = 100% white; center = 0%, blue). Tumors were segmented into 4 layers along the tumor radius (1 = 0–25%; 2 = 25–50%; 3 = 50–75%; 4 = 75–100% of the tumor radius). Acquisitions were performed by using 1× optical objective and 1× numeric zoom. To measure distance from tumor center and vessels, each cell was dotted with the spot tool according to the local intensity contrast and shortest distance from tumor center or vessels. Each CD45^+^ cell is represented by one spot statistically coded by the shortest distance from vessels (Fig. S9 and Movies S1 and S2). CD45^+^ cells were classified in 3 categories in function of their distance from vessels (1 = 0-10 µm, 2 ≥ 10–50 µm, 3 ≥ 50). Acquisitions were performed by using 4× optical objective and 2.5× numeric zoom. The same brains were used for all these analyses. 3D reconstitution and image analyses were performed by using Imaris software.

#### Two-photon imaging

Hemi-beads were removed from images and only horizontal plans were considered. Spectral unmixing was first applied to raw two-photon images (Zen software) and the mean cell density (number of cells/mm^3^) was calculated over tumoral volume using Imaris software v10.0 (Bitplane). The volume of each tumor was defined by creating a 3D mask using the surface tool from Imaris. Only cells present in the tumor mask were segmented according to the local intensity contrast. Cells found in blood vessels were easily excluded thanks to a 3D mask associated to the vasculature. Hence, to access the different cell densities, the reported number of LysM-EGFP^+^, CD11c-EYFP^+^ and LysM-EGFP^+^/CD11c-EYFP^+^ cells was reported over the tumor volume. Colocalization analyses were performed using Imaris software v10.0 (Bitplane) to identify LysM-EGFP^+^/CD11c-EYFP^+^ double-labeled cells.

### Statistical analyses

Categorical variables were presented as percentages, continuous variables as mean and standard error of the mean (sem). The Chi-square test (or Fisher’s exact test) was used to compare categorical variables. To test the differences in means of two independent series, the parametric t-test and the non-parametric Wilcoxon–Mann–Whitney test were used. To compare mean spheroid size variations according to different treatments, over time, we performed the analysis of variance (ANOVA) test, and after confirmation of normality, the means were compared by the ANOVA post-hoc Tukey test. For prognostic value analysis, overall survival (OS) was defined to be time from the date of surgery to death, censored at the date of last contact. The Kaplan–Meier method was used to estimate survival distributions. Log-rank tests were used for univariate comparisons. For CYTOF experiments, percent of total and mean intensities of marker expression were calculated per populations for each timepoint. Data were visualized using Tableau Desktop Software 2023.1. Mean intensities were transformed in asinh and centered to the mean. OPLS-DA analysis was applied on the dataset using SIMCA16 Multivariate analysis software (Sartorius) to find variables correlated with defined experimental groups of samples. All the tests were 2-sided and *p* < 0.05 was considered significant for each statistical analysis (*p*-value; *p-value < 0,05; **p-value < 0.005; ***p-value < 0,0005; ****p-value < 0,0001). Statistical analyses were conducted using the the GraphPad Prism 5.0 statistical software.

## Supplementary information

Supplementary data

## Data Availability

The data underlying Figs. 3, 4, 5, 6 are openly available by using the following link: https://figshare.com/projects/Melanoma-inhibitor_of_apoptosis_protein_a_key_driver_of_microglia_phenotype_and_glioblastoma_immune_microenvironment/187641. The data underlying Fig. 7 are available through cytobank: https://inserm.cytobank.org/cytobank/experiments/39026/illustrations/1; https://inserm.cytobank.org/cytobank/experiments/38619/illustrations/18; https://inserm.cytobank.org/cytobank/experiments/38747; https://inserm.cytobank.org/cytobank/experiments/39057. The data underlying Fig. 1 are openly available at the following link: 10.5281/zenodo.6962901.
