# Supplementary material for: SMAC mimetic drives microglia phenotype and glioblastoma immune microenvironment
Source: Cell Death Dis. 2024 Sep 15;15(9):676. doi: 10.1038/s41419-024-07056-z (PMC11402972; doi:10.1038/s41419-024-07056-z)
Supplement: Supplementary file 1 — Supplementary data [file 41419_2024_7056_MOESM1_ESM.pdf]

SMAC mimetic drives microglia phenotype and glioblastoma immune microenvironment

Emmanuel Snacel-Fazy[1,2], Aurélie Soubéran[1,2,3], Magali Grange[4], Kevin Joseph[5,6,7,8], Carole Colin[1,2], Philippe Morando[1,2], Hervé Luche[4], Alessandra Pagano[1], Sophie Brustlein[9], Franck Debarbieux[10], Soline Toutain[1,2], Carole Siret[11], Serge A. van de Pavert[11], Geneviève Rougon[10], Dominique Figarella-Branger[1], Vidhya Madapusi Ravi[5,6,7,8], Emeline Tabouret[1,3,12] and Aurélie Tchoghandjian[1,2 *]

Supplementary Material and Methods

Single-cell RNA sequencing

scRNAseq analysis was carried out using a publicly available integrated dataset from the Glioblastoma microenvironment (https://doi.org/10.1101/2022.08.27.505439, Ruiz-Moreno, Cristian. GBmap. (2022) doi:10.5281/zenodo.6962901.). This dataset was composed by the integration of 16 datasets, with 338,564 cells from a total of 110 patients. The dataset comprised of 127,521 neoplastic cells and 211043 cells from the local microenvironment, all annotated at multiple levels.

The data was analyzed, and visualizations were created out using the Seurat package for single cell RNAseq data [49-52]. Dotplots of gene expression across all cellular populations was carried out using the "Dotplot" function implemented within Seurat. Kernel based visualization of gene expression of interest within cellular populations was carried out using the "plot_density" function, included within the Nebulosa package for R [53]. All analysis was carried out in the R computing environment.

GL-261-Dsred and CT2A cell culture

GL261-DsRed [17] and CT2A cells (Sigma-Aldrich, Paris, France ; newly purchased) were cultured as previously described To grow as spheroids, 5000 cells were cultured per well in

96-well plats U-bottom with a supplement of 20% of methylcellulose for 24 h. Mycoplasma detection was regularly performed during the time of the experimentations.

ML-IAP silencing by siRNA technology

Cells were transfected with lipofectamine 2000 (ref. 11668027, Thermoficher), diluted in OPTIMEM (ref. 31985062, Gibco) and with the different siRNA targeting ML-IAP at the concentration of 40 nM. The sequences were the following: si-RNA 1 (5'-GACCTAAAGACAGTGC CAAGTGCCT-3' ; exon 1 position 197-222 pb), si-RNA 2 (5'-GAAGAGACTTTGTCC ACAGTGTGCA-3' ; exon 3 position 668-693 pb), si-RNA 3 (5'-CCTGGTCTGTGCTGAG TGT-3' ; exon 6 position 999-1018 pb), si-RNA 4 (5'-GGAAGAGACTTTGTCCACA-3' ; exon 3 position 667-686). After 24 hours, the expression of ML-IAP was analyzed in cell protein lysats by western blotting.

Explant cultures

Explant cultures were performed as previously described [54]. Five GB tissue samples were collected after surgery and placed in HBSS. Tissues were cut into 500 µm pieces with a tissue chopper (Phymep) and plated on 24-well plates precoated with poly-(L)-lysine (10 µg/ml; Sigma-Aldrich). Explants were cultured in a serum-free medium composed of DMEM/F12 medium supplemented with hormones, anti-biotics and growth factors as previously described [47] at 37°C in a humidified atmosphere of 5% CO_2 and 95% air including 20% O_2. After 7-10 days of culture, explants were treated with vehicle (DMSO) or 1 µM of SMg for 72h. Then explants were washed with PBS and fixed with paraformaldehyde 4% for immunostainings.

Glioma mouse models

One hundred mouse glioma GL261-DsRed cells were implanted in the cortex of 7 weeks-old C57BL/6 mice as already described [17]. Briefly, 50.000 cells/µl were automatically injected into the cerebral cortex (-1 mm anterior to bregma, − 1 mm lateral and − 1 mm in deep of the cortex surface) for a final volume of 2 µl over 10 minutes. A round glass coverslip (diameter:

5mm) was then sealed on the adjacent bone and fixed to the skull by dental cement. Animals were observed until they fully recovered. The body weight and clinical status of mice were recorded every 2 days. Mice were sacrificed when they exhibited >20% reduction from initial body weight or significant neurological deficit. Mice were weekly treated with GDC-0152 (SMg, intravenously, 20 mg/kg in solution in DMSO [24]). Treatment started 1 week after cell graft (Fig. S3). For immunofluorescences and clearing, mice were anesthetized, brains were extracted and fixed 24h in 4% paraformaldehyde at 4°C until use. For immunofluorescences, brains were cut in the coronal axis in 50 µm thick slices with a vibratome. For immunohistochemistry, brains were fixed with 4% formol, dehydrated and embedded in paraffine.

For two-photon imaging LysM-EGFP [55], CD11c-EYFP [56], and Thy1-CFP [57] (JAX stock #003710) mice were crossed to obtain triple transgenic mice [17]. Spheroids of GL261-DsRed cells were injected as previously described [17]. A Sephadex hemi-bead with diameter fitting with the dura-mater opening was inserted in the injection wound and glued using histo-compatible acrylic glue (Cyanolit). For immunofluorescences, mice were anesthetized, brains were extracted and fixed 4 h in paraformaldehyde 4% and then freeze until use.

Whole mount stainings and Clearing

Hemi brains were processed following the iDISCO+ protocol as previously described [58]. Shortly, brains were dehydrated in successive methanol baths, put in a solution of 66% dichloromethane and bleached with 5% H_2O_2. Then samples were re-hydrated in sequential baths of methanol and permeabilized (PBS/0.2% TritonX-100/20% DMSO/0.3M glycine) at 37°C for 2.5 days. Samples were the incubated in a blocking solution (PBS/0.2% TritonX-100/10% DMSO/3% Donkey Serum) at 37°C for 4.5 days and incubated for 5 days in primary antibodies solution (see Table S2; PBS-Tween 0.2% with heparin 10 mg/mL (PTwH)/5% DMSO/3% donkey serum) at 37°C for 5 days. After washing, samples were incubated with

secondary antibodies (see Table S2) in PTwH/3% donkey serum at 37°C for 5 days. Samples were finally dehydrated in successive baths of methanol and cleared in a BABB solution (33% Benzyl alcohol, 66% Benzyl benzoate) for 24 h and then with BABB alone until light sheet acquisition.

Tumoroids were cleared by using the MACS clearing kit according to the manufacturer protocol (Miltenyi Biotec).

Two-photon imaging

Images acquisition was conducted as reported [17] at D21 and D28 (Fig. S6). Briefly, prior to each imaging session, mice were anesthetized and injected intravenously with 100 µL of a quantum dots (QDots) solution (Qtracker™ 705 Vascular Labels, ThermoFisher, 6 µg/100 µL in phosphate saline buffer, Sigma Aldrich) and positioned on a stereotaxic frame allowing movements in the three-directions. Repositioning of the mice at D28 was realized using visual vascular landmarks.

We used a Zeiss LSM 780-MP two-photon microscope home modified to allow animal positioning below the 20X water immersion objective (1.0 NA) and coupled to a femtosecond pulsed infrared tunable laser (Chameleon Ultra 2, Coherent). Images were acquired using an excitation wavelength tuned at 920 nm to excite all fluorophores simultaneously. Signals were epicollected and separated by dichroic mirrors and filters on five independent non-descanned detectors. Gains and offsets were identical for all the detectors, except for the red channels whose gain was reduced by 30% to compensate for the strong expression of DsRed in tumor cells. Images were acquired below the dura matter over a depth of 500 µm using 10 µm steps. Laser power was linearly increased with depth. Z-stack images were acquired as mosaics (stitching mode) to cover the whole tumor surface.

Immunostainings

For immunofluorescences, a 2 h blockage of non-specific sites was firstly realized (bovine serum albumin 5%, goat serum 5%, donkey serum 5%, Triton 0.3%). Primary antibodies (Supp Table 1) were then incubated overnight at 4°C in PBS/BSA 5%/Triton 0.3%. Secondary antibodies (Supp Table 1) were incubated 1 h at room temperature with Hoechst. Slices were incubated with copper sulfate/ammonium chloride for 10 min and then slices were mounted with Prolong Mounting Medium (Sigma-Aldrich). Acquisitions were performed with a Zeiss LSM700 or LSM780 confocal microscope.

CD31 immunohistochemistry was performed by using a benchmark automate (Ventana, Roche) as previously described[24]. Stained slices were scanned with a Hamamatzu Nanozoomer and visualized with the NDP.view2 software. Ten areas of the tumors were analyzed at the objective 20x. Size of the larger diameter of vessels was manually drown and number of vessels was counted.

Protein extraction and western blotting

Western blots were performed as previously described[24]. Proteins were extracted with RIPA lysis buffer supplemented with proteases and phosphatases inhibitors for 30 minutes (Thermo Scientific) on ice followed by 10 minutes centrifugation at 12000rpm. Protein concentration was assayed using bicinchoninic acid (MicroBCA kit, Pierce, Rockford, IL, USA). Eighty micrograms of proteins per lane were separated by 12% (for IAP proteins detection) or 4-20% (for cell signaling pathway proteins) Mini-PROTEAN® TGX™ Precast Gels (Biorad). Proteins were transferred onto nitrocellulose membranes (Novex) using wet transfer system (Biorad) at 100V for 1 h. After 1 h of blocking in 5% skimmed milk and 3 washes with TBS Tween 0.1% (TBST, Sigma-Aldrich), membranes were incubated with the antibodies (see table 1) in TBST supplemented with 5% BSA (Sigma-Aldrich) overnight at 4°C under shaking. The following horseradish peroxidase-conjugated donkey anti-mouse IgG, goat anti-rabbit IgG (Santa Cruz Biotechnology) antibodies and ECL (Bio-Rad) were used for proteins detection.

Quantifications were performed using ImageJ software (National Institutes of Health, Bethesda, MD, USA) on preflashed X-ray films. Data presented were standardized on β-actin expression and for phospho-proteins, quantifications are represented by phospho-protein/total-protein ratio. The original western blots are presented in Fig. S11 and Fig. S12.

Cell viability assay

Cell viability was evaluated by assessing cell metabolic capacity using the MTT method (3-(4,5-dimethylthiazol-2yl)-diphenyl tetrazolium bromide; Sigma-Aldrich). Cells were treated with serial concentrations of SMg (0.1, 1, 10, 25, 50, 100, 150, 200, 500 µM). After treatment, MTT reagent was added and incubated for 4 h at 37 °C. The reduced formazan was dissolved with DMSO and absorbance was measured at 562 nm with an Elx800 microplate reader (Bio-Tek, Colmar, France) and data were analyzed with Gen5 1.09 software (Bio-Tek).

TCGA analyses

Transcriptomic data using Affymetrix Human Genome U133A Array were extracted from the open access Cancer Genome Atlas (TCGA) database[59]. The data of 357 patients with primary tumor diagnosed as Glioblastoma, *IDH*-wt were analyzed using the GlioVis data portal[60] to determine the prognostic value of the mRNA expression of the following genes: *CD14*, *CCL17* and *SALL1*. Data of BIRC3 and P2RY13 mRNA expression were also extracted to be analyzed as ratios (BIRC3/P2RY13).

Supplementary Figures S1 to S12

Fig. S1. Experimental workflow from human GB samples.

Fig. S2. Effect of IAP inhibition on human immune cells.

(A) Immunofluorescence of labeled $CD45^+$ cells (green) and nuclei (Hoechst, blue) after CD45 immunomagnetic cell sorting from human GB samples (scale bar=100 µm). **(B)** Histograms representing the purity of the CD45 cultures isolated from human GB samples 7 days after

sorting. One representative experiment out of 6 is shown. **(C)** Graphs representing quantification of cytokines/chemokines differentially expressed in the supernatant of vehicle and SMg-treated (1 µM) CD45 cultures. **(D)** Survival curve obtained from TCGA databases of GB patients showing correlation between *CD14* and *CCL17* expression and patient overall survival. **(E)** One representative fluorescent activated cell sorting experiment out of 4 illustrating the TMEM119 cell sorting.

Fig. S3. IAP inhibition affects TAM-MG function.

(A) Viability assay of GL261-DsRed spheroids upon SMg treatment (n= 3). **(B)** Viability assay of CT2A spheroids upon SMg treatment (n= 3). **(C)** MTT viability assay of C8B4 cells upon SMg treatment (n=2). **(D)** Quantification of IAP expression levels in C8B4 microglia cell line after 72h of vehicle or SMg treatment from 3 independent experiments. ImageJ software was used. Data presented were normalized to actin β expression. **(E)** Quantification of CT2A spheroids area upon ZVAD pre-treatment + vehicle (n=33), ZVAD pre-treatment + SMg (n=29), TNFαi pre-treatment + vehicle (n=32), TNFαi pre-treatment + SMg (n=35) and in the presence (vehicle, n=32; SMg, n=34) or absence of the C8B4 cells (vehicle, n=48; SMg, n=48). The spheroids sizes were normalized to the size measured after 24 h. Statistical analyses were performed by using ANOVA test and ANOVA post-hoc Tukey test; alpha=0.05, bilateral p-value: ** = p<0.005.; ****=p<0.0001. **(F)** Quantification of GL261-DsRed spheroids area (mm^2) upon vehicle (n=5) and SMg treatment (n=6) and with or without conditioned medium upon vehicle (n=5) and SMg treatment (n=5). Statistical analyses were performed by using ANOVA test; alpha=0.05. **(G)** Representative western blot of ML-IAP expression in siCTRL and siML-IAP C8B4 cells after 24h of siRNA treatment. ML-IAP expression was analyzed by western blotting Expression level of Actin β served as loading control. **(H)** Quantification of GL261-DsRed spheroids area (mm^2) upon vehicle (n=5) and SMg treatment (n= 6) and with or without conditioned medium upon vehicle (n=5) and SMg treatment (n= 5). C8B4 cells were

pretreated for 24h with ZVAD. Statistical analyses were performed by using ANOVA test; alpha = 0.05. Statistical analyses were performed by using ANOVA test; alpha=0.05.

A, B, C, D, E, F, H Bar graphs represent mean ± s.e.m.

Fig. S4. Experimental workflow of the experiments performed by using a syngeneic glioma mouse model.

Fig. S5. SMg decreases tumor growth and promotes immune infiltration.

(A) Representative 3D reconstitution of cleared hemi-brains at 15, 21 and 28 days-post tumor graft (D15, D21 and D28) in vehicle (n=11) and SMg-treated (n=10) mice. Tumor cells were labelled with an anti-RFP antibody (Red) and brains were outlined in white (scale bar = 2000µm). **(B)** Tumor area for vehicle (D15 n= 4, D21 n= 3 and D28 n=4) and SMg (D15 n= 4, D21 n= 4 and D28 n=3) condition. Statistical analyses were performed by using Mann-Whithney test; alpha = 0.05, bilateral p-value: ¤: p=0.057. **(C)** Tumor area representing the growth of tumors in vehicle (n=6) and SMg-treated mice (n= 6) followed at D15, D21 and D28 with epifluorescence miscroscopy. Statistical analyses were performed by using Mann-Whithney test; alpha = 0.05, bilateral p-value: *: p<0.05. **(D)** Two-photon images of D21 and D28 GL261-DsRed tumors (scale bar = 300 µm). Representative images are shown. **(E)** Illustration of the 4 layers representing the distance from tumor center (from tumor center: 0-25%, 25-50%,50-75% and 75-100%). Tumor border is outlined in green (scale bar=500 µm). **(F)** Histogram count plots representing the repartition curve of $CD45^+$ cell quantity (y-axis) from tumor center (0 on the x-axis) to tumor border (right extremity on the x-axis) of D28 vehicle and SMg-treated tumors. Dotted blue line shows $CD45^+$ cells distribution in number of spots, dashed blue line represents random distribution and red lines represent attraction distance.

B, C Bar graphs represent mean ± s.e.m.

A, D, E 3D reconstitution and image analyses were performed by using Imaris software.

Fig. S6. IAP inhibition remodels tumor vasculature.

(A) Two-photon images of vessels (white) and tumor (red) in vehicle and SMg-treated mice at D21 and D28 (scale bar, D21=200 µm; D28=300 µm). White dotted squares identify zoomed areas. **(B)** Scatter plot representing vessel area at D28 in vehicle (n=9) and SMg (n=3) treated tumors. Statistical analyses were performed with Mann-Whitney test; alpha=0.05, bilateral p-value: *:<0.05; **: p<0.005. **(C)** CD31 immunolabeling of formalin fixed paraffin embedded tumor slices (scale bar=250 µm). Representative images are shown. A higher magnification is presented in the lower panel. **(D)** Scatter plot representing vessels area and the number of vessels at D28 in vehicle (n= 11) and SMg (n= 10) treated tumors. Statistical analyses were performed by using Mann-Whitney test; alpha = 0.05, bilateral p-value: ns: p> 0.05, bilateral p-value: ****: p< 0.0001. **(E)** 3D reconstitutions of cleared hemi-brains at D15, D21 and D28 in vehicle (n= 11) and SMg-treated (n=10) mice, vessels are represented in white (scale bar=50 µm). A 4X zoom was applied. Representative images are shown.

A, E 3D reconstitutions and image analyses were performed by using Imaris software.

B, D Bar graphs represent mean ± s.e.m.

Fig. S7. SMg modifies the sphericity and the cell surface area of the TAMs.

(A) Confocal images of $EGFP^+$ (green) and $Ly6G^+$ (purple) cells in vehicle and SMg treated tumors at D28. GL261-DsRed tumor cells are in red (scale bar=100 µm). Representative images are shown. **(B)** Quantification of $EGFP^+/Ly6G^+$ cells in vehicle (n=5) and SMg (n=6) treated tumors at D28. Bar graphs represent mean ± s.e.m. Statistical analyses were performed with Mann-Whitney test; alpha = 0.05. **(C)** Two-photon images representing cells with high (0.900) and low (0.600) sphericity scores related to vessels (white). Scale bar=10µm. **(D)** Scatter plot representing the distance from vessels (x-axis, µm) in function of the morphology represented by a sphericity score (y-axis, score from 1=Round, 0 = not round) at D21 ($EGFP^+$: vehicle n=5152, SMg n=1058; $EYFP^+$: vehicle n=6920; SMg n=2481; LysM-$EGFP^+$/CD11c-$EYFP^+$

:vehicle n=1332, SMg n=547) and D28 ($EGFP^+$: vehicle n=15268, SMg n=14398; $EYFP^+$: vehicle n=11483, SMg n = 7194; $EGFP^+$/ $EYFP^+$: vehicle n=4267, SMg n=4581). Each dot represents a cell. Linear regressions are represented for each graph. **(E)** Scatter plot representing the distance from vessels (x-axis, μm) in function of the area (y-axis, μm^2) at D21 ($EGFP^+$: vehicle n=5152; SMg n=1058; $EYFP^+$: vehicle n=6920; SMg n=2481; $EGFP^+$/$EYFP^+$: vehicle n=1332 ; SMg n=547) and D28 ($EGFP^+$: vehicle n=15268, SMg n=14398; $EYFP^+$: vehicle n=11483 ; SMg n=7194; $EGFP^+$/$EYFP^+$: vehicle n=4267, SMg n=4581). Each dot represents a cell. Linear regressions are represented for each graph.

C, D, E 3D reconstitution and image analyses were performed by using Imaris software.

Fig. S8. SMg reprograms GB immune landscape.

(A) Multivariate data analysis by SIMCA of all the 5 experiments of vehicle (D21 n= 22, D28 n= 4) SMg-treated (D21 n= 23, D28 n=6) mice. **(B)** Proportional sizes of the respective immune cell clusters projected onto a *t*-SNE map. **(C)** Heat map of the different immune cell clusters projected onto a *t*-SNE map of vehicle and SMg-treated tumors of the other experiment performed at D21. **(D)** Pie charts demonstrating the distribution of the identified immune cell types across conditions of the D. **(E)** Heat map of the different immune cell clusters projected onto a *t*-SNE map of vehicle and SMg-treated tumors of Exp A at D28. **(F)** Pie charts demonstrating the distribution of the identified immune cell types across conditions of the E. **(G)** Percentage of basal and active TAM-MG expressing PD1 among the $CD45^+$ cells at D21 (basal TAM-MG n=3; active TAM-MG n=3) and D28 (basal TAM-MG n=5; active TAM-MG n=5). Vehicle and SMg treatments were pooled. Bar graphs represent mean ± s.e.m. Statistical analyses were performed by using Mann-Whitney test; alpha = 0.05, bilateral p-value: $**=p<0.005$.

Fig S9. Images processing pipeline.

Fig S10. Tumor segmentation based on 488 wavelength contrast.

Fig S11. Original western blots of Fig. 1 and 3. (A) Western blots corresponding to the Fig. 1 A. (**B)** Western blots corresponding to the Fig. 3 A. (**C)** Western blots corresponding to the Fig. 3 F-H.

Fig S12. Original western blots of Fig. S.3. (A) ML-IAP expression. **(B)** Actinβ expression.

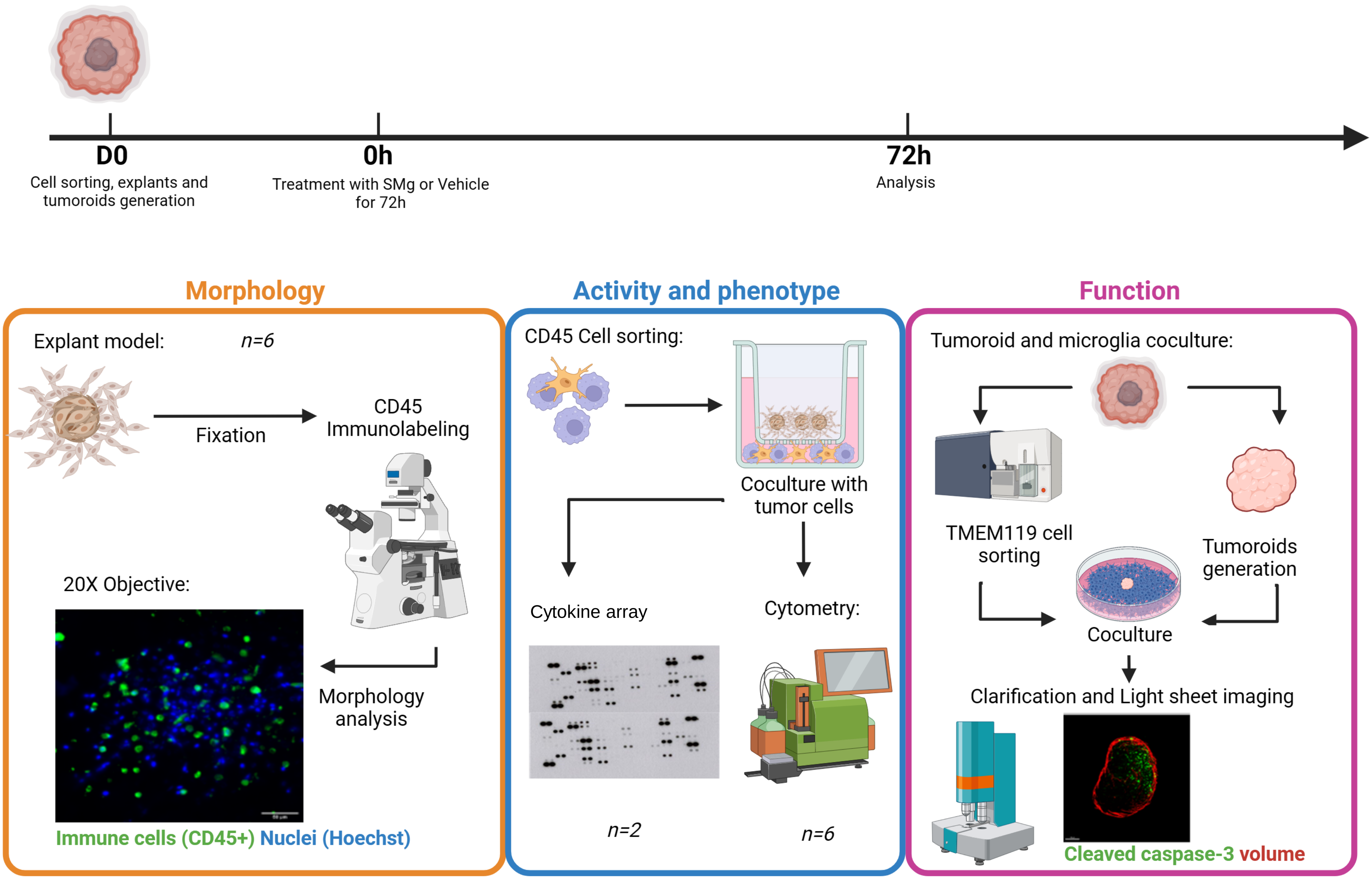

Fig. S1. Experimental workflow from human GB models.

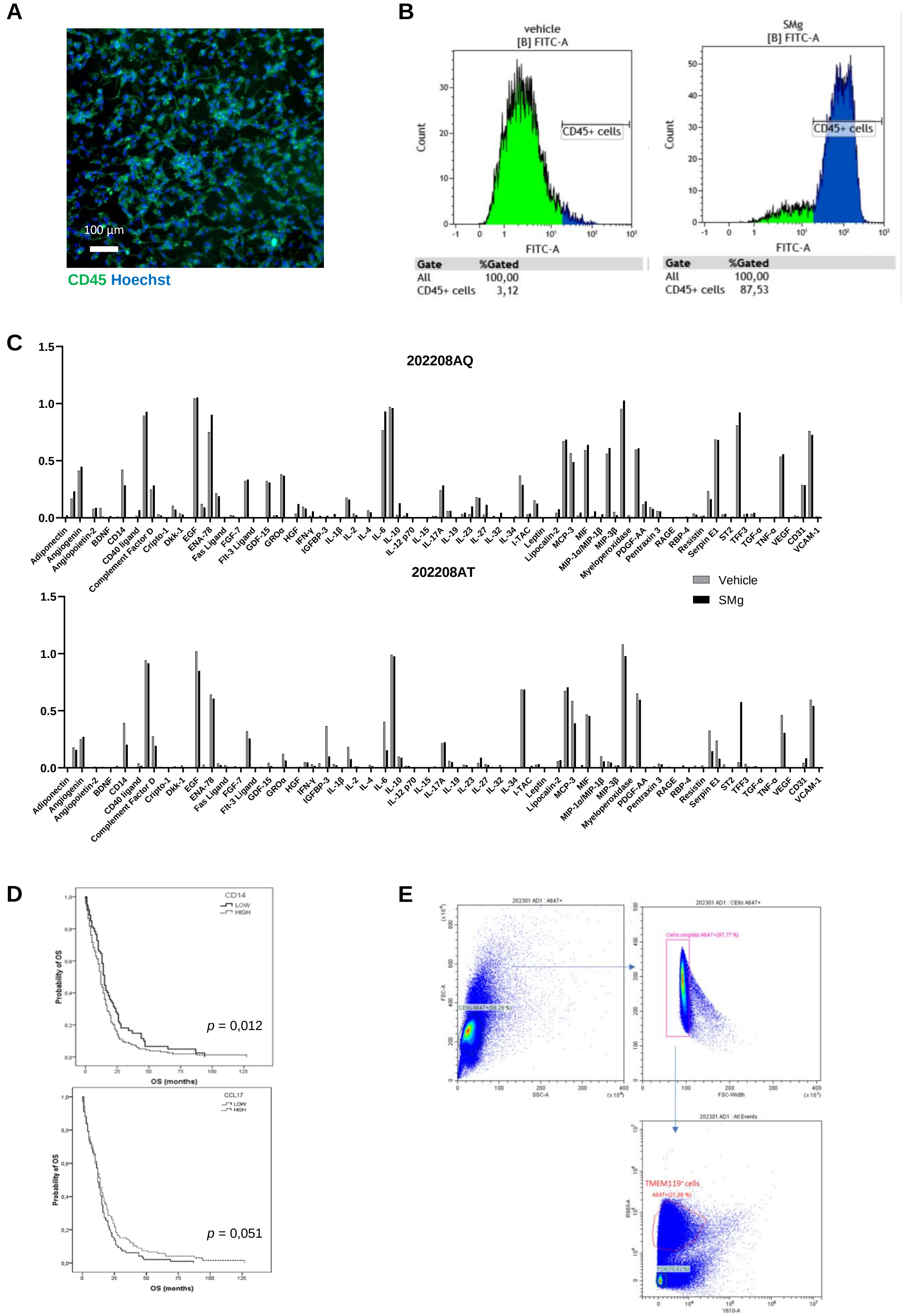

Fig. S2. Effect of IAP inhibition on human immune cells.

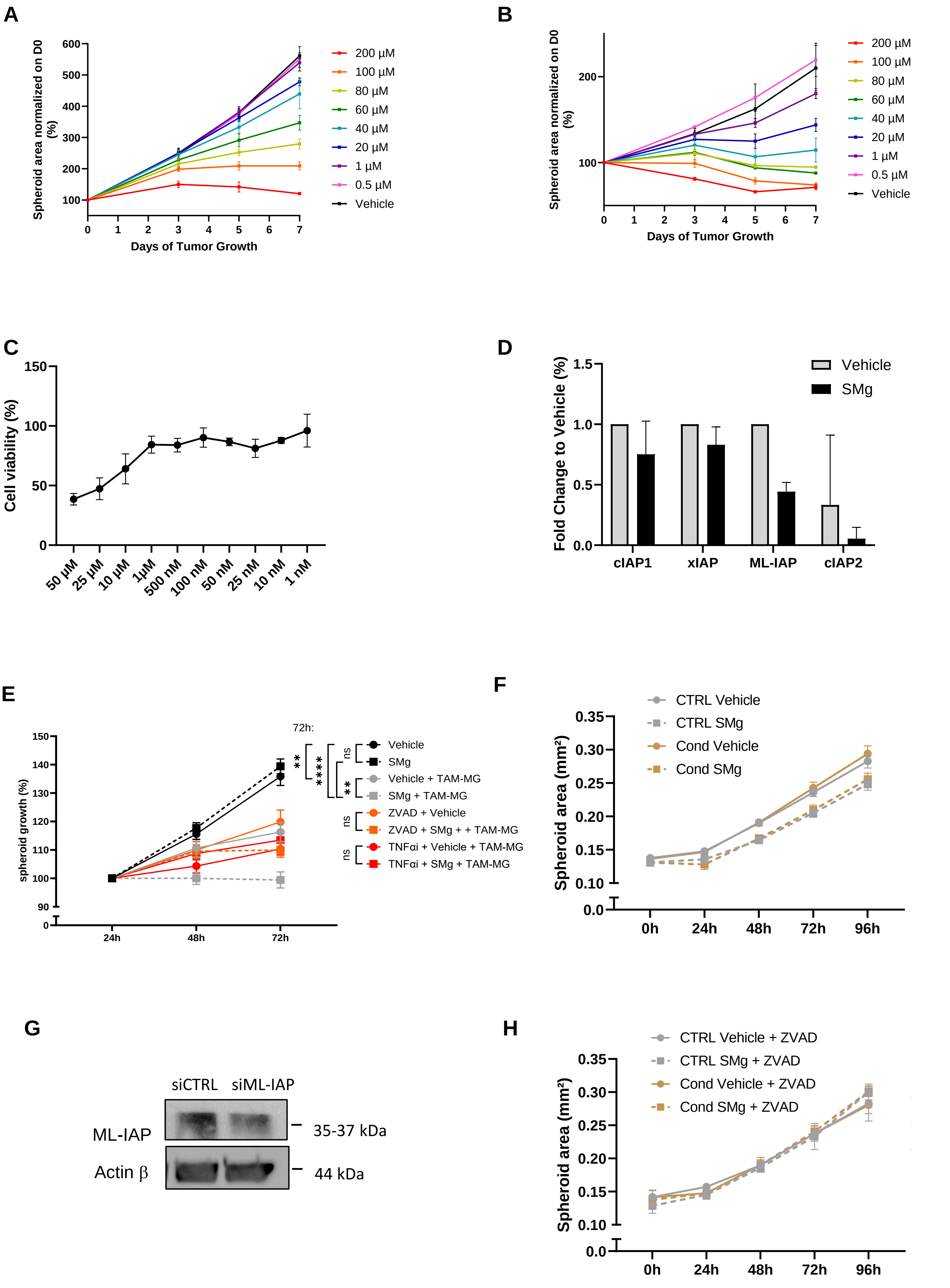

Fig. S3. IAP inhibition affects TAM-MG function.

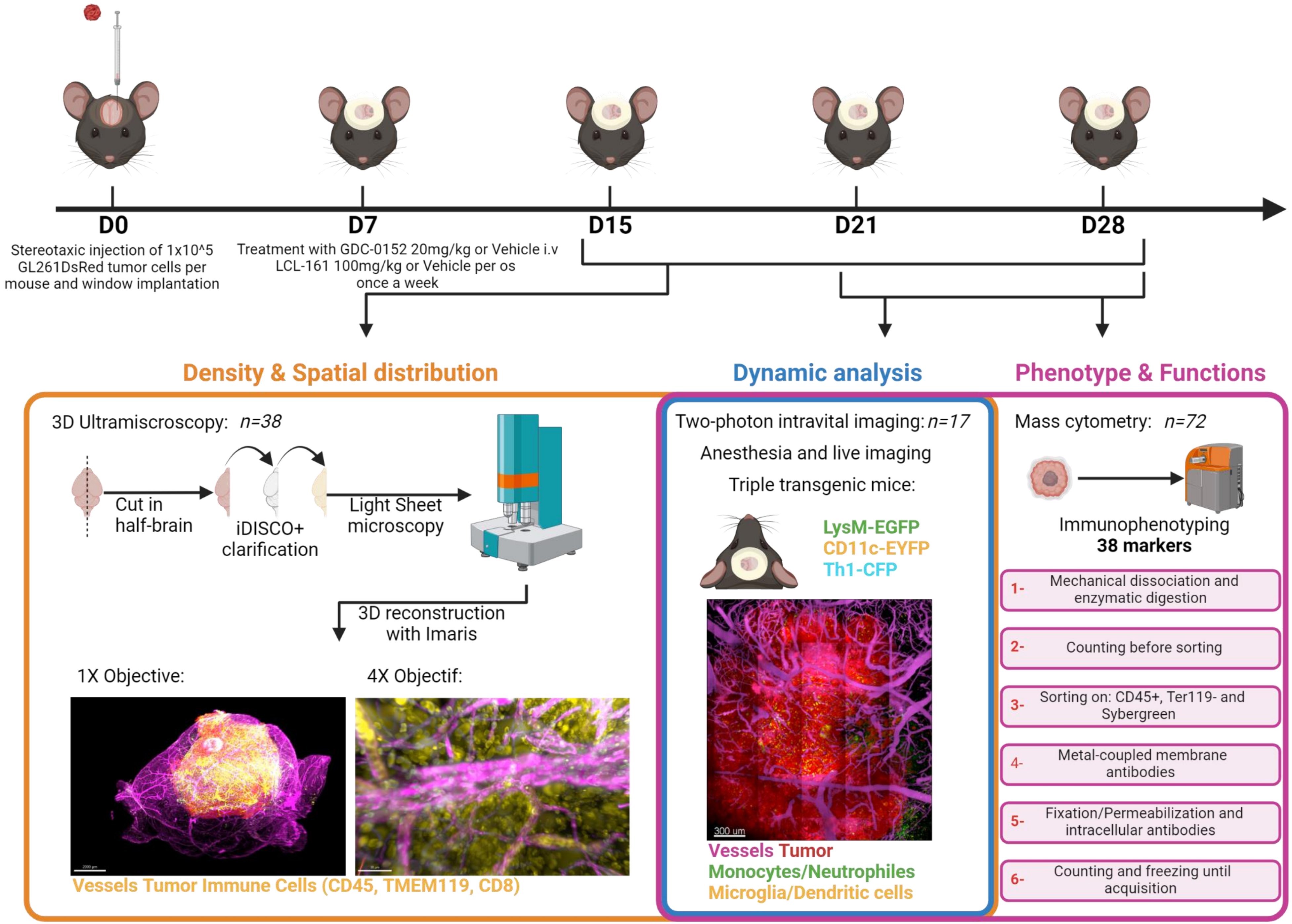

Fig. S4. Experimental workflow of the experiments performed by using a syngeneic glioma mouse model.

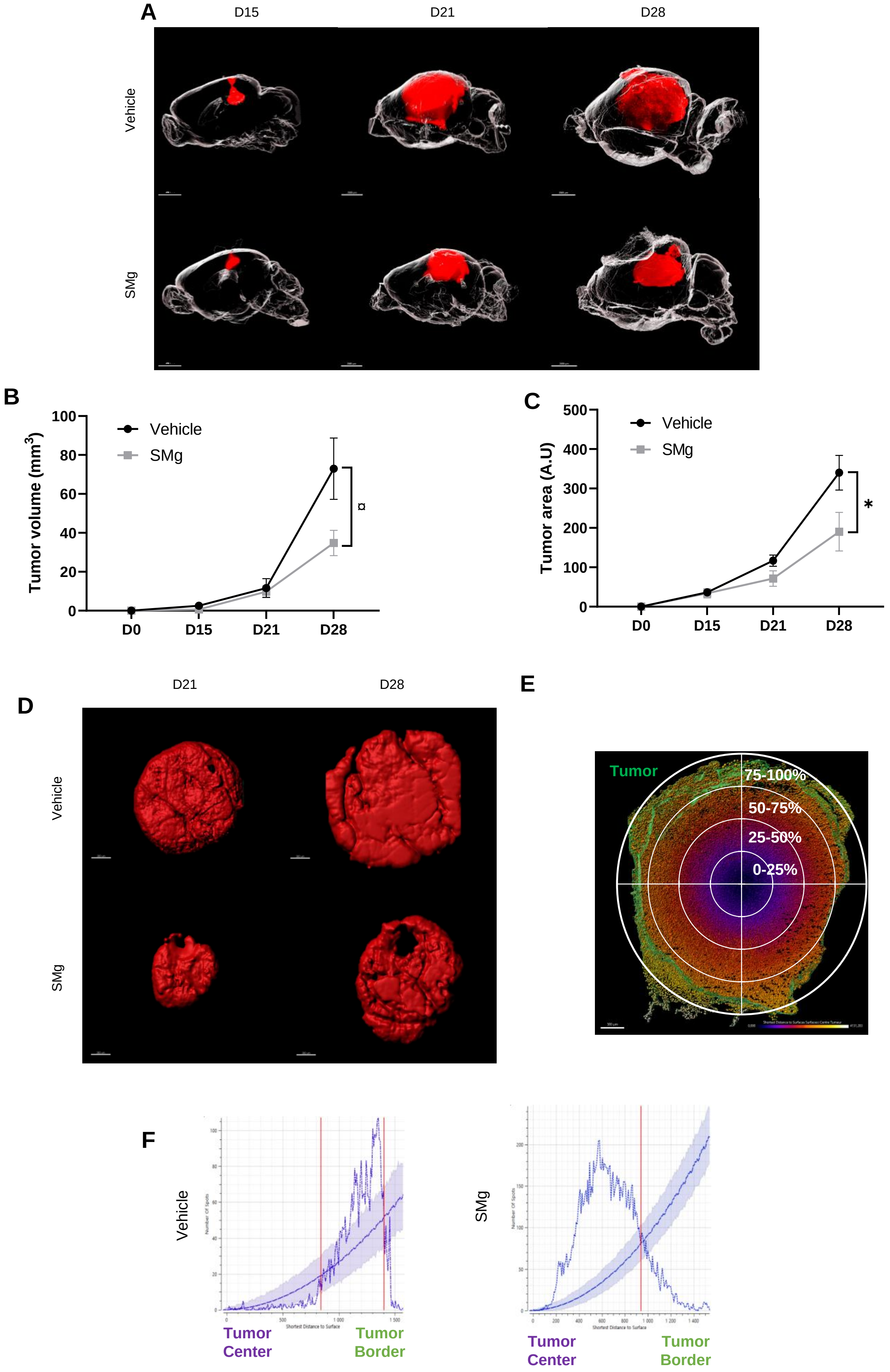

Fig. S5. SMg decreases tumor growth and promotes immune infiltration.

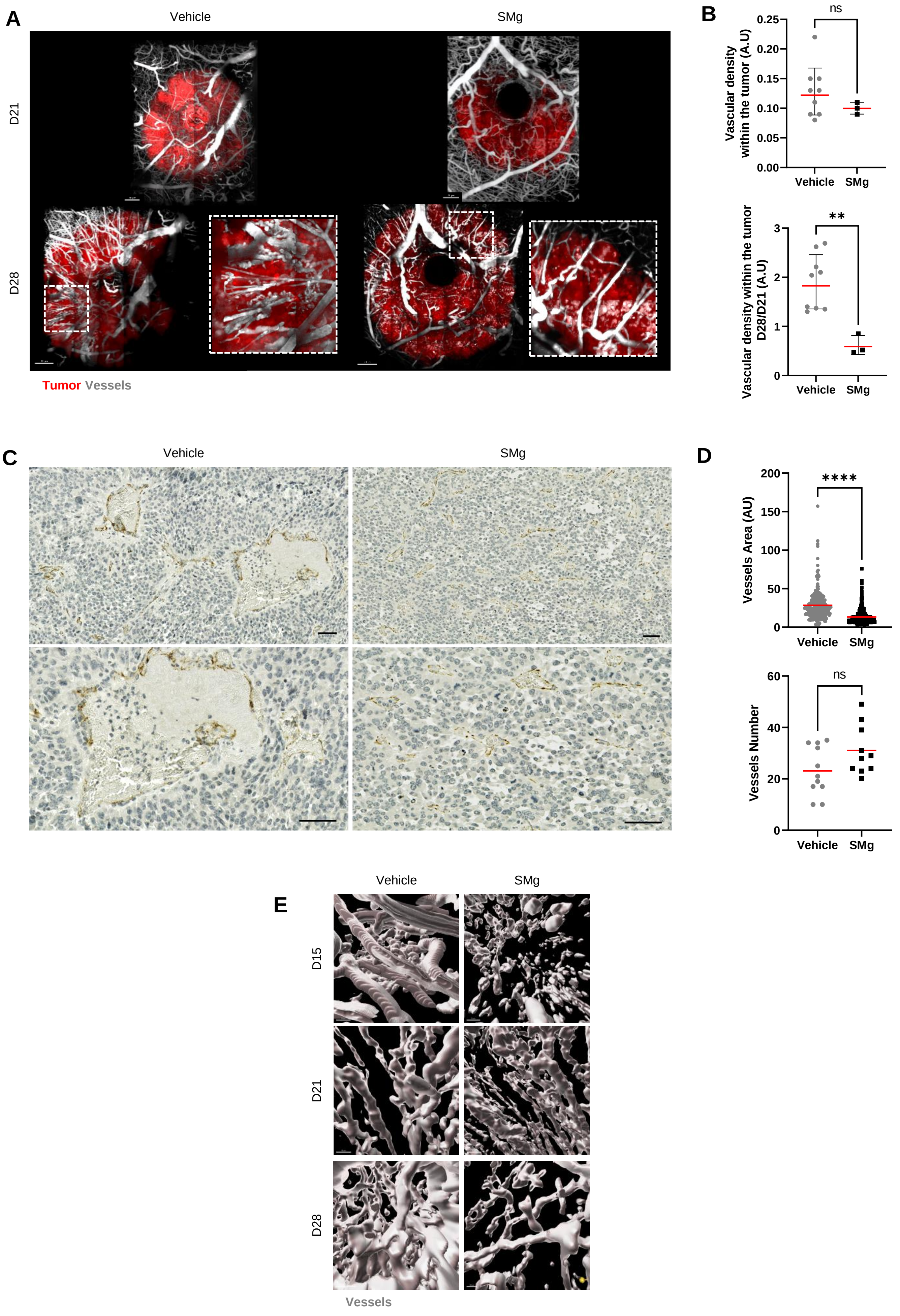

Fig. S6. IAP inhibition remodels tumor vasculature.

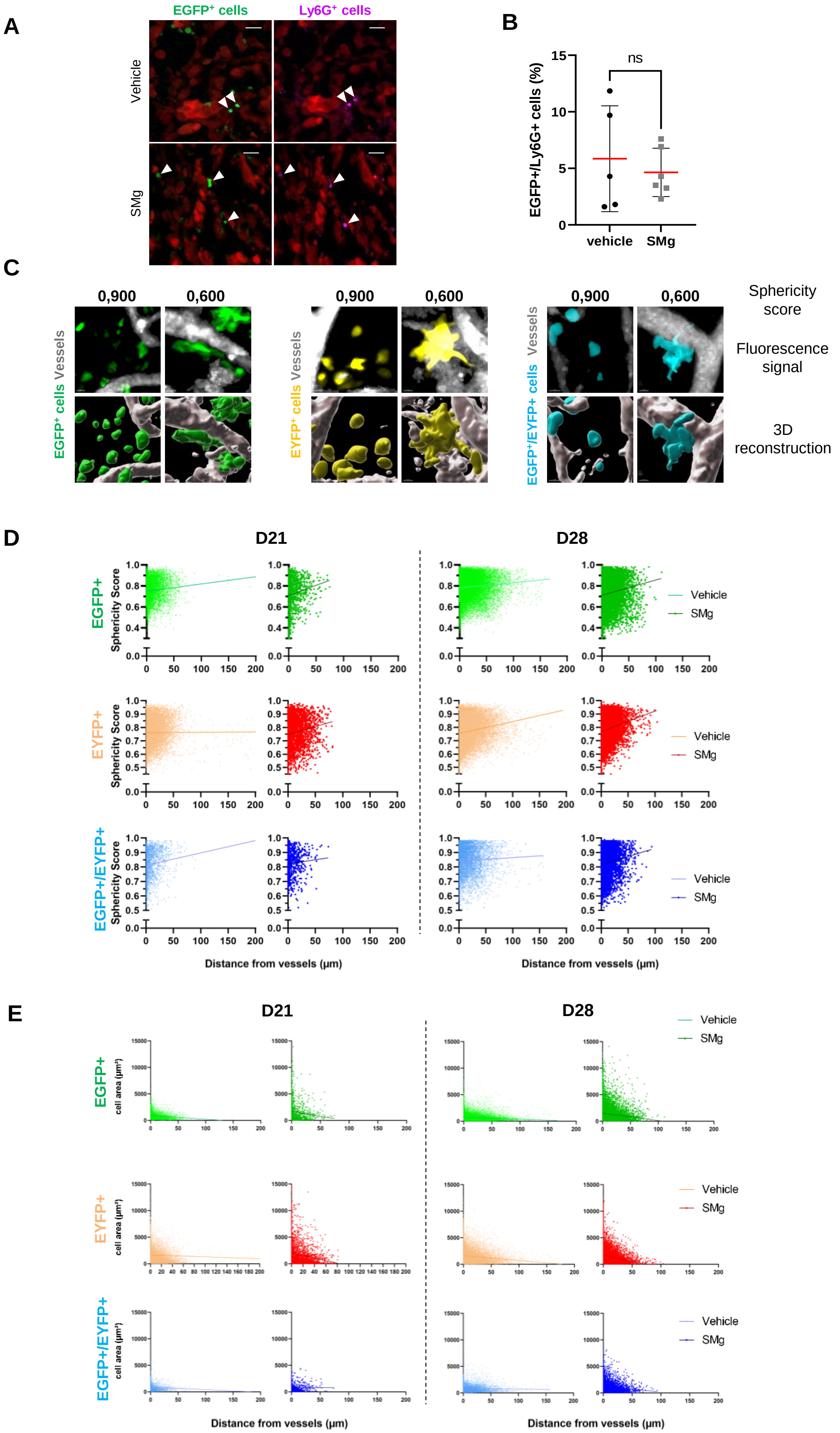

Fig. S7. SMg modifies the sphericity and the cell surface area of the TAMs.

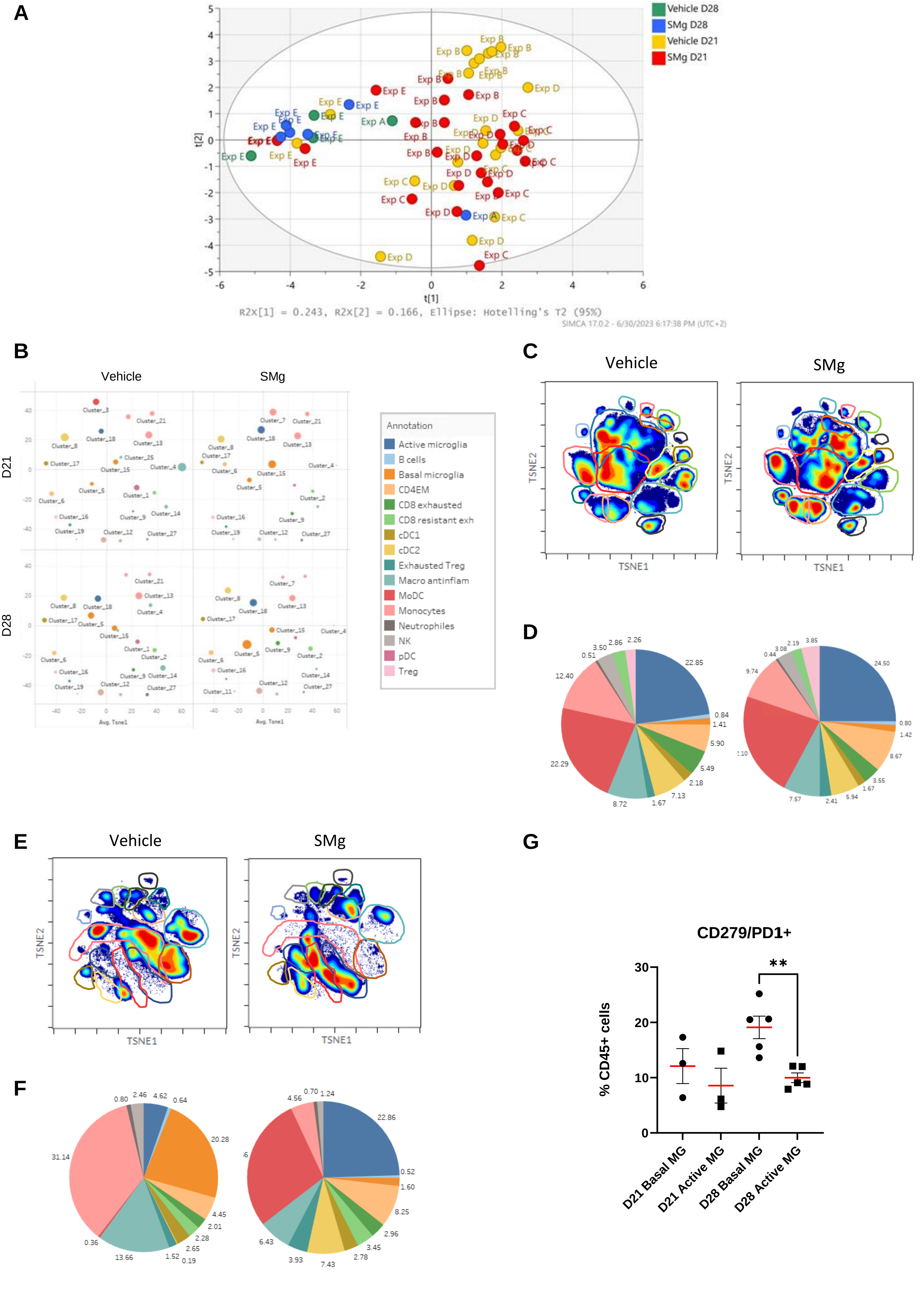

Fig. S8. SMg reprograms GB immune landscape.

A Image treatment

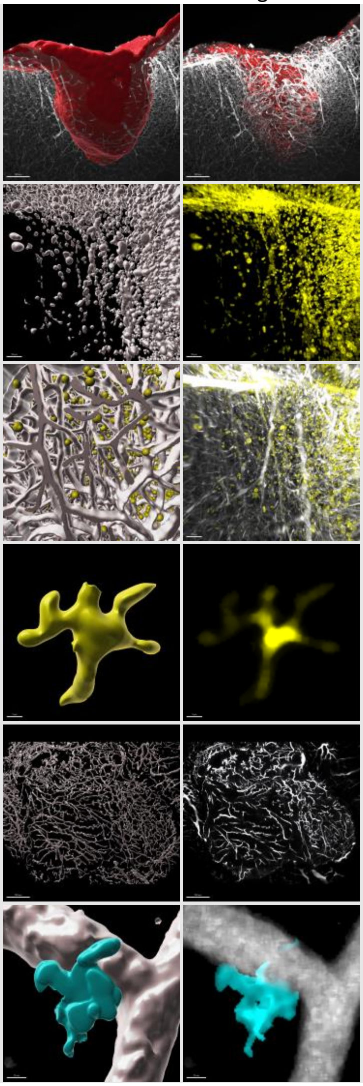

Imaris tool	Extracted data
**Surface**	Tumor volume
**Surface**	Number of voxels<br>Number of CD45$^+$ cells
**Surface - Spot**	Distance between immune cells and vessels<br>Distance between CD45$^+$ cells and tumor center<br>Number of TMEM119$^+$ cells in contact with CD8$^+$ cells
**Surface**	Sphericity score<br>Area
**Surface**	Vessel surface<br>Vessel density
**Surface - Surface**	Distance between EGFP$^+$/EYFP$^+$ cells and vessels

B Data analysis

Ultramicroscopy

Parameter	Unit
Tumor volume	*μm^3*
Number of voxels	*Voxels*
Number of CD45$^+$ cells	-
Distance between immune cells and vessels	*μm*
Distance between CD45$^+$ cells and tumor center	*μm*
Distance between TMEM119$^+$ cells and CD8$^+$ cells	*μm*

Two-photon imaging

Parameter	Unit
Sphericity score	-
Area	*μm^2*
Vessel surface	*μm^2*
Vessel density	*$\mu m^2/\mu m^3$*
Distance from vessels for EGFP$^+$/EYFP$^+$ cells	*μm*

Fig S9. Images processing pipeline.

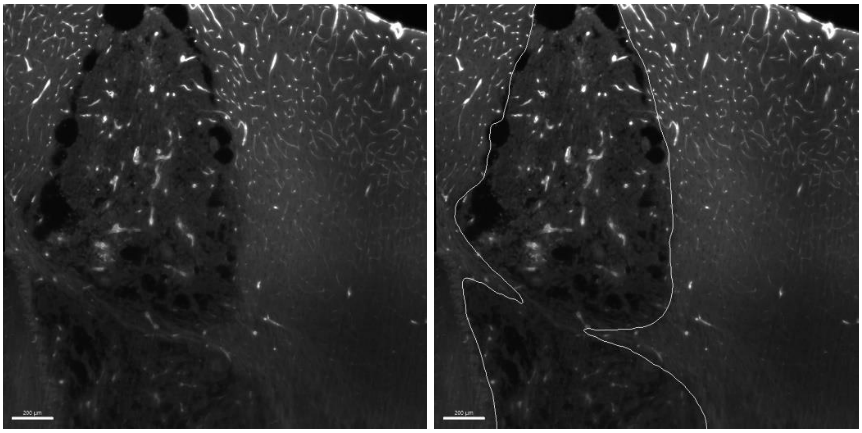

Vessels + Contrast (488 wavelength) | Vessels + Contrast (488 wavelength) + tumor segmentation

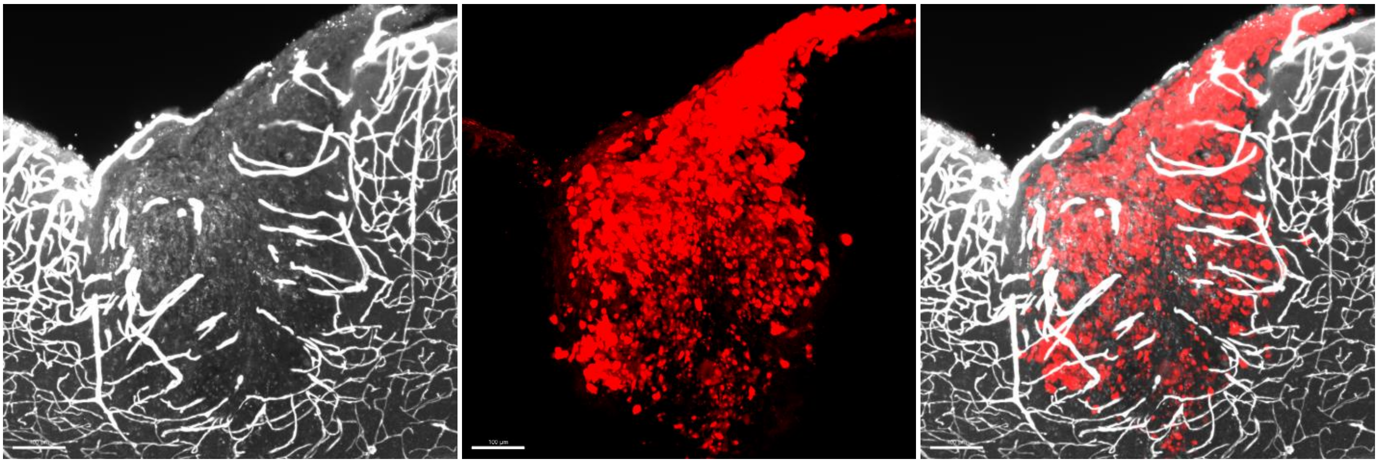

Vessels + Contrast (488 wavelength) | GL261DsRed | Vessels + Contrast (488 wavelength) + GL261DsRed

Fig S10. Tumor segmentation based on 488 wavelength contrast

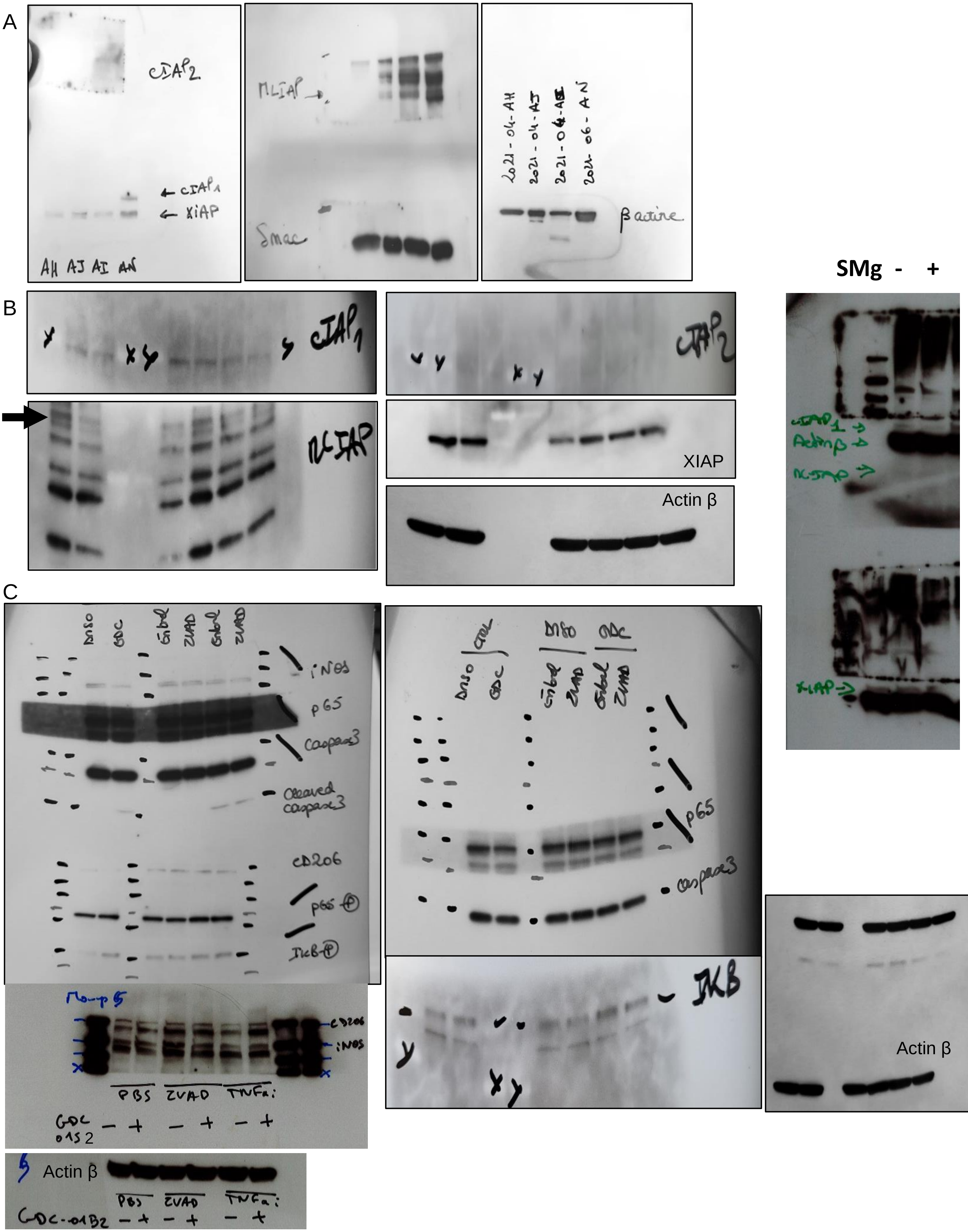

Fig. S11: Original western blots

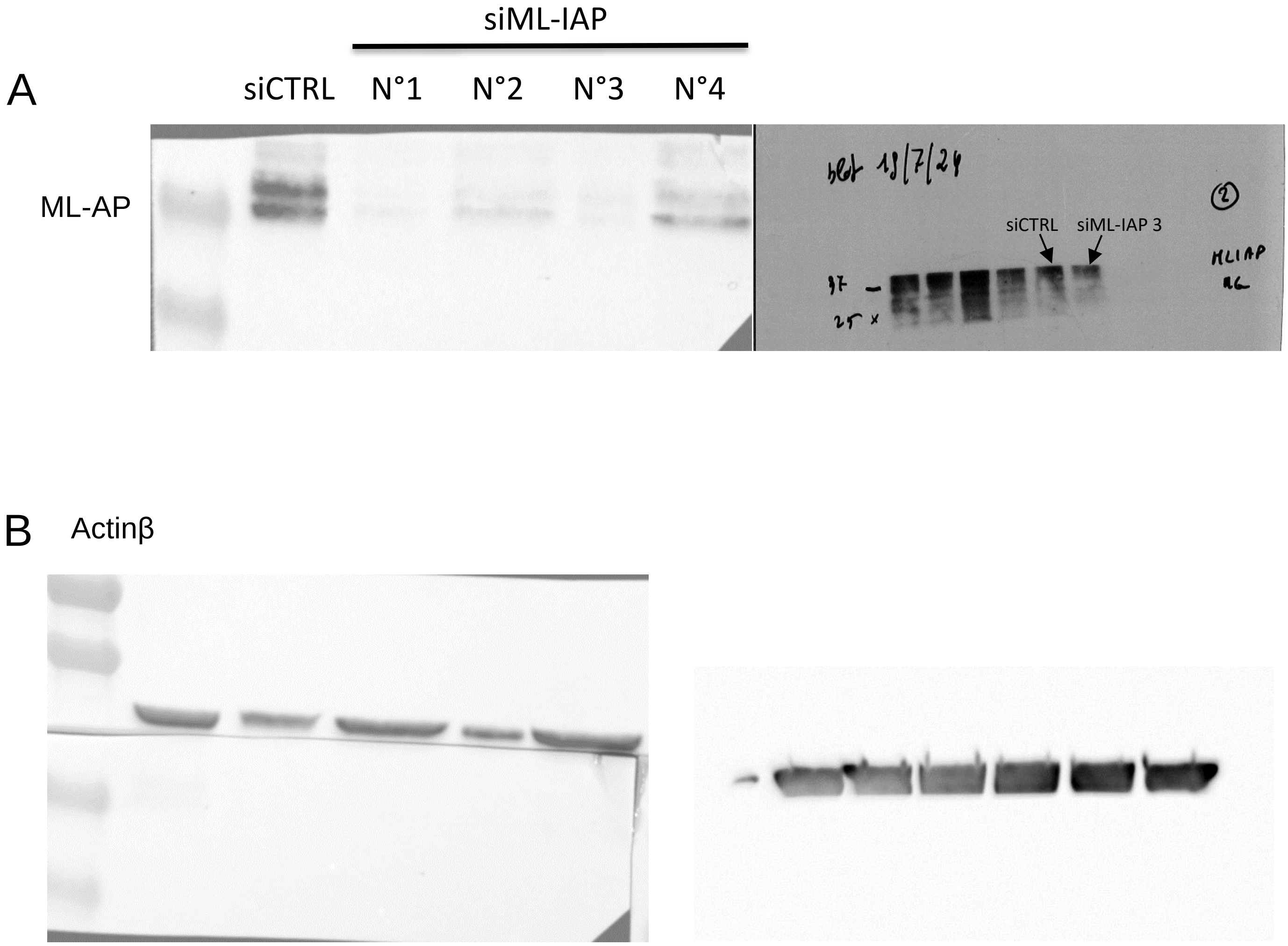

Fig. S12: Original western blots of Fig. S3.

Table S1: Patient characteristics and clinical data.

Characteristics	N	%
**Age** (median, range), years	68 (32 - 82)	
**Gender** (women / men)	7 / 12	*37 / 63*
**Histology**		
Glioblastoma, *IDH*-wild-type	18	*95*
Astrocytoma, *IDH*-mutated, grade 3	1	*5*
***MGMT* promoter**		
Unmethylated	11	*58*
Methylated	7	*37*
NA	1	*5*
**Surgery**		
Gross total resection	11	*58*
Partial resection	6	*31*
Surgical biopsy	2	*11*
**Karnofsky Performans Status at diagnosis**		
< 70	5	*27*
70	7	*37*
80	6	*31*
NA	1	*5*
**First line treatment**		
Radio-chemotherapy	17	*89*
Chemotherapy alone	2	*11*

Table S2: Antibodies panel.

Antibody	Host	Reactivity	Clone	Conjugated	Mono/polyclonal	Isotope	Experiment	Dilution	Supplier	Ref	Home-made
Anti-Ly6G	Rat	Mouse	RB6-8C5	Unconjugated	monoclonal	IgG2b	Immunochemistry	1:50	Invitrogen	14-5931-82	No
Anti-Laminine	Rabbit	Mouse	-	Unconjugated	polyclonal	IgG	Immunochemistry	1:100	Novus bio	NB300-144	No
Anti-TMEM119	Rabbit	Mouse/ Human	E3E10	Unconjugated	monoclonal	IgG	Immunochemistry	1:100	Cell Signaling	90840	No
Anti-MHCII	Rabbit	Mouse	M5/114.15.2	Unconjugated	monoclonal	IgG2b	Immunochemistry	1:200	Invitrogen	14-5321-82	No
Anti-RFP	Rabbit	Mouse	-	Unconjugated	polyclonal	IgG	Whole mount	1:2000	Rockland	600401379	No
Anti-CD45	Rat	Mouse	30-F11	Unconjugated	monoclonal	IgG2b	Whole mount	1:200	Santa Cruz	SC-53665	No
Anti-CD31	Goat	Mouse	-	Unconjugated	polyclonal	IgG	Whole mount	1:700	R&D	AF3628	No
Anti-Podocalixin	Goat	Mouse	-	Unconjugated	polyclonal	IgG	Whole mount	1:700	R&D	AF1556	No

Anti-αSMA	Goat	Mouse	-	Unconjugated	polyclonal	IgG	Whole mount	1:700	Abcam	ab21027	No
Anti-TMEM119	Rabbit	Mouse	28-3	Unconjugated	monoclonal	IgG	Whole mount	1:700	Abcam	ab209064	No
Anti-CD8a	Human	Mouse	REA601	Vio667	monoclonal	IgG1	Whole mount	1:50	Miltenyi Biotec	130-129-594	No
Anti-Smac	Mouse	Mouse/ Human	56/Smac/DIABLO	Unconjugated	monoclonal	IgG	Western Blot	1:1000	BD Biosciences	612246	No
Anti-MLIAP	Mouse	Mouse/ Human	88C570	Unconjugated	monoclonal	IgG1k	Western Blot	1:500	Novus bio	NB100-56548	No
Anti-xIAP	Mouse	Mouse/ Human	-	Unconjugated	polyclonal	IgG1	Western Blot	1:1000	BD Biosciences	610716	No
Anti-cIAP1	Rabbit	Mouse/ Human	D5G9	Unconjugated	monoclonal	IgG	Western Blot	1:1000	Cell Signaling	7065	No
Anti-cIAP2	Rabbit	Mouse/ Human	-	Unconjugated	polyclonal	IgG	Western Blot	1:1000	Merk	AB3615	No
Anti-β actine	Mouse	Mouse/ Human	AC-15	Unconjugated	monoclonal	IgG1	Western Blot	1:5000	Sigma	A5441	No
Anti-Caspase 3	Rabbit	Mouse/ Human	A5D175	Unconjugated	Rabbit	IgG	Western Blot	1:1000	Cell Signaling	9661	No

P-p65	Rabbit	Mouse/ Human	S536	Unconjugated	monoclonal	IgG	Western Blot	1:100	Cell Signaling	3033	No
P65	Mouse	Mouse/ Human	F-6	Unconjugated	monoclonal	IgG	Western Blot	1:50	Santa Cruz	Sc-8008	No
iNOS	Rabbit	Mouse/ Human	D6B6S	Unconjugated	monoclonal	IgG	Western Blot	1:1000	Cell Signaling	13120	No
Anti-CD45	Mouse	Mouse/ Human	30-F11	Unconjugated	monoclonal	IgG2b	Immunochemistry	1:200	Invitrogen	14-0451-82	No
Anti-CD45	Mouse	Human	HI30	FITC	monoclonal	IgG1	Flow Cytometry	1:10	BD pharmagen	560976	No
Anti-CD206	Mouse	Human	DCN228	PE	monoclonal	IgG1	Western Blot	1:50	Miltenyi Biotec	130-124-233	No
Anti-CD11c	Human	Human	REA618	PEVio770	monoclonal	IgG1	Flow Cytometry	1:50	Miltenyi Biotec	130-113-588	No
Anti-TMEM119	Rabbit	Human	106-6	AF647	monoclonal	IgG	Flow Cytometry	1:100	Abcam	Ab225494	No
Anti-IA/IE	Rat	Human	M5/114.15.2	APCCy7	monoclonal	IgG2b	Flow Cytometry	1:100	Biolegend	107627	No
Anti-Rat	Donkey	Rat	-	Cy5	polyclonal	IgG	Immunochemistry	1:100	Jackson Immunoresearch	712–175-150	No

Anti-Rabbit	Goat	Rabbit	-	AlexaFluor 488	polyclonal	IgG	Immunochemistry	1:1000	Invitrogen	A11011	No
Anti-Rabbit	Donkey	Rabbit	Poly4084	AlexaFluor 647	polyclonal	IgG	Immunochemistry	1:1000	Biolegend	406414	No
Anti-Rat	Donkey	Rat	MRG2b85	AlexaFluor 647	monoclonal	IgG1	Immunochemistry/ Brain clearing	1:1000 / 1:600	Biolegend	408209	No
Anti-Rabbit	Donkey	Rabbit	-	AlexaFluor 555	polyclonal	IgG	Whole mount	1:600	Invitrogen	A31572	No
Anti-Goat	Donkey	Goat	-	AlexaFluor 488	polyclonal	IgG	Whole mount	1:600	Invitrogen	A11055	No
Anti-CD45.2	Mouse	Mouse	104	111 Cd	monoclonal	IgG2a	Mass Cytometry	-	Biolegend	109802	Yes
Anti-CD45.2	Mouse	Mouse	104	112 Cd	monoclonal	IgG2a	Mass Cytometry	-	Biolegend	109802	Yes
Anti-CD45.2	Mouse	Mouse	104	114 Cd	monoclonal	IgG2a	Mass Cytometry	-	Biolegend	109802	Yes
Anti-CD45.2	Mouse	Mouse	104	116 Cd	monoclonal	IgG2a	Mass Cytometry	-	Biolegend	109802	Yes
Anti-CD45.2	Mouse	Mouse	104	110 Cd	monoclonal	IgG2a	Mass Cytometry	-	Biolegend	109802	Yes

Anti-CD45.2	Mouse	Mouse	104	106 Cd	monoclonal	IgG2a	Mass Cytometry	-	Biolegend	109802	Yes
Anti-CD11b	Rat	Mouse	M1/70	115In	monoclonal	IgG2b	Mass Cytometry	-	Biolegend	101202	Yes
Anti-CD11c	Armenian Hamster	Mouse	N418	172Yb	monoclonal	IgG	Mass Cytometry	-	Biolegend	117341	Yes
Anti-Ly6G	Rat	Mouse	1A8	141Pr	monoclonal	IgG2a	Mass Cytometry	1:100	Fluidigm	3141008 B	No
Anti-CD39	Rat	Mouse	24DMS1	142Nd	monoclonal	IgG2a	Mass Cytometry	1:100	Fluidigm	3142005 B	No
Anti-CD274	Rat	Mouse	10F.9G2	144Nd	monoclonal	IgG2b	Mass Cytometry	-	Biolegend	124302	Yes
Anti-CD4	Rat	Mouse	RM4-5	145Nd	monoclonal	IgG2a	Mass Cytometry	-	Biolegend	100505	Yes
Anti-CD8a	Rat	Mouse	53-6.7	146Nd	monoclonal	IgG2a	Mass Cytometry	-	Biolegend	100746	Yes
Anti-IRF8	Mouse	Mouse	V3GYWCH	147Sm	monoclonal	IgG1	Mass Cytometry	-	Invitrogen	53-9852-82	Yes
Anti-CX3CR1	Mouse	Mouse	SA011F11	148Nd	monoclonal	IgG2a	Mass Cytometry	-	Biolegend	149002	Yes

Anti-IRF4	Rat	Mouse	IRF4.3E4	149Sm	monoclonal	IgG1	Mass Cytometry	-	Biolegend	646402	Yes
Anti-Ly6C	Rat	Mouse	HK1.4	150Nd	monoclonal	IgG2c	Mass Cytometry	-	Biolegend	128002	Yes
Anti-CD64	Mouse	Mouse	X54-5/7.1	151Eu	monoclonal	IgG1	Mass Cytometry	1:100	Fluidigm	3151012 B	No
Anti-CD25	Rat	Mouse	3C7	151Eu	monoclonal	IgG2b	Mass Cytometry	1:100	Fluidigm	3151007 B	No
LysM	Mouse	Mouse	MA1-82873	152Sm	monoclonal	IgG2a	Mass Cytometry	-	Invitrogen	BGN-0696-5B1	Yes
Anti-CD152	Hamster	Mouse	UC10-4B9	154Sm	monoclonal	IgG	Mass Cytometry	1:100	Fluidigm	3154008 B	No
Anti-CD279	Rat	Mouse	29F.1A12	155Gd	monoclonal	IgG2a	Mass Cytometry	-	Biolegend	135202	Yes
Anti-CD192	Rat	Mouse	SA203G11	156Gd	monoclonal	IgG2b	Mass Cytometry	-	Biolegend	93501	Yes
Anti-CD68	Rat	Mouse	FA-11	158Gd	monoclonal	IgG2a	Mass Cytometry	-	Invitrogen	14-0681-82	Yes
Anti-CD73	Rat	Mouse	TY11.8	159Tb	monoclonal	IgG1	Mass Cytometry	-	Biolegend	127202	Yes

Anti-FoxP3	Rat	Mouse	MF14	161Dy	monoclonal	IgG2b	Mass Cytometry	-	Biolegend	126402	Yes
Anti-TNFα	Rat	Mouse	MP6-XT22	162Dy	monoclonal	IgG1	Mass Cytometry	1:100	Fluidigm	3162002 B	No
Anti-Klrg1	Syrian hamster	Mouse	2F1	163Dy	monoclonal	IgG	Mass Cytometry	-	Invitrogen	16-5893-85	Yes
Anti-CD172a	Rat	Mouse	P84	164Dy	monoclonal	IgG	Mass Cytometry	-	BD Bioscience	51-410-28671	Yes
Anti- MER-TK	Human	Mouse	REA477	165Ho	monoclonal	IgG1	Mass Cytometry	-	Miltenyi Biotec	130-107-477	Yes
Anti-CD26	Rat	Mouse	H194-112	166Er	monoclonal	IgG2a	Mass Cytometry	-	Biolegend	137801	Yes
Anti-F4/80	Rat	Mouse	BM8	167Er	monoclonal	IgG2a	Mass Cytometry	-	Biolegend	123102	Yes
Anti- XCR1	Mouse	Mouse	ZET	168Er	monoclonal	IgG2b	Mass Cytometry	-	Biolegend	148202	Yes
Anti-CD206	Rat	Mouse	C068C2	169Tm	monoclonal	IgG2a	Mass Cytometry	1:100	Fluidigm	3169021 B	No
Anti-CD161	Mouse	Mouse	PK136	170Er	monoclonal	IgG2a	Mass Cytometry	1:100	Fluidigm	3170002 B	No

Anti-CD44	Rat	Mouse	IM7	171Yb	monoclonal	IgG2b	Mass Cytometry	1:100	Fluidigm	3171003B	No
Anti-CD317	Rat	Mouse	129c1	173Yb	monoclonal	IgG2b	Mass Cytometry	-	Biolegend	127102	Yes
Anti-Gata3	Mouse	Mouse	16E10A23	175Lu	monoclonal	IgG2b	Mass Cytometry	-	Biolegend	653802	Yes
Anti-CD62L	Rat	Mouse	MEL-14	160Gd	monoclonal	IgG2a	Mass Cytometry	1:100	Fluidigm	3160008B	No
Anti-CD3e	Armenian hamster	Mouse	145-2C11	176Yb	monoclonal	IgG	Mass Cytometry	-	Biolegend	100302	Yes
Anti-IA IE	Rat	Mouse	M5/114.15.2	209Bi	monoclonal	IgG2b	Mass Cytometry	-	Fluidigm	3209006B	No
Anti-CD38	Rat	Mouse	90	153Eu	monoclonal	IgG2a	Mass Cytometry	-	Biolegend	102702	Yes
Anti-CD88	Rat	Mouse	20,7	174Yb	monoclonal	IgG2b	Mass Cytometry	-	Biolegend	135802	Yes
Anti-Eomes	Mouse	Mouse	7C9B03	143Nd	monoclonal	IgG1	Mass Cytometry	-	Biolegend	662002	Yes
Anti-Iba1	Rabbit	Mouse	E4O4W	113In	monoclonal	IgG	Mass Cytometry	-	Cell signaling	17198B	Yes

Anti-CD45.2	Mouse	Mouse	104	APC	monoclonal	IgG2a	Mass Cytometry	1:1000	Biolegend	109814	No
Anti-Ter119	Rat	Mouse	TER119	PE	monoclonal	IgG2b	Mass Cytometry	1:100	BD pharmagen	553673	No
SytoxGreen	-	-	-	-	-	-	Mass Cytometry	-	Invitrogen	S7020	No
